# Supplementary material for: Moiré effect enables versatile design of topological defects in nematic liquid crystals
Source: Nat Commun. 2024 Feb 26;15:1655. doi: 10.1038/s41467-024-45529-z (PMC10897219; doi:10.1038/s41467-024-45529-z)
Supplement: Supplementary file 1 — Supplementary Information [file 41467_2024_45529_MOESM1_ESM.pdf]

Moiré effect enables versatile design of topological defects in nematic liquid crystals

Supplementary Information

Xinyu Wang[1†], Jinghua Jiang[2†], Juan Chen[2,3†], Zhawure Asilehan[2], Wentao Tang[1], Chenhui Peng[2‡], Rui Zhang[1*]

[1]Department of Physics, The Hong Kong University of Science and Technology, Clear Water Bay, Kowloon, Hong Kong, China
[2]Department of Physics, University of Science and Technology of China, Hefei, Anhui 230026, China
[3]Department of Physics and Materials Science, The University of Memphis, Memphis, Tennessee 38152, USA

[†]equal contribution
[‡]cpeng2@ustc.edu.cn
[*]ruizhang@ust.hk

Contents

1 1D cusp-like splay-bend pattern 2
1.1 Mapping from nematic moirés to isotropic moirés 2
1.2 Theoretical prediction of defects in the thin-film limit 2
1.3 Understanding the defect state diagram 3
1.4 The details of the nematic structure 5
1.5 Periodicity and tilting angle of 1D geometric moiré and 1D nematic moiré . . . 8
1.5.1 $(1, -1)$-moiré 8
1.5.2 $(1, 1)$-moiré 11
1.5.3 T, T' and ω in the nematic moiré 12

2 1D sinusoidal splay-bend pattern 13
2.1 Defect structure prediction in thin-film limit 13
2.2 The details of the nematic structure 15
2.3 Defect configurations at different rotation angle Ψ 15

3 2D defect lattice 17
3.1 Cell gap effect 17
3.2 Defect configurations at different rotation angles Ψ 17
3.3 The details of the nematic structure for $\Psi = 5°$ 19
3.4 2D geometric moiré theory 21
3.5 Director field at $\Psi = 36.8°$ 25
3.6 The effect of rotation center 25
3.7 Statistics of the quasi-loops 25

4 Reconfiguration of nematic moiré pattern under an electric field 29
4.1 Frederiks transition in the twist cell 29
4.2 The Frederiks transition in the nematic moiré 29
4.3 The responses at different angles 31

5 Additional details of the methods 31
5.1 Experiment details 31
5.2 Simulation details 35
5.2.1 Modeling details 35
5.2.2 Initial director fields in the simulations 37
5.2.3 The rotation speed for fast rotation simulations 38
5.2.4 Effects of LC elastic constants 41
5.2.5 Simulation method for generating POM images 41

1 1D cusp-like splay-bend pattern

1.1 Mapping from nematic moirés to isotropic moirés

The anisotropic nature of liquid crystals gives richer texture in nematic moiré than in isotropic moiré. For 1D cusp-like and 1D sinusoidal splay-bend pattern, the variation of the surface-preferred director $\mathbf{n}_s$ along the x-coordinate in a period L is shown in Fig. S1(top row). θ is the orientation angle of the unit vector $\mathbf{n}_s$. Angle θ_g represents the corresponding black-white pattern. Different from the angle θ, θ_g varies between $0°$ (mapped to the white color) and $90°$ (mapped to the black color), and doesn't distinguish clockwise rotation from counter clockwise rotation, $\theta_g = |(|\theta - 90°| - 90°)|$. Through angle θ and θ_g, the director field pattern $\mathbf{n}_s$, equivalently represented by a colored bar (Fig. S1 middle row), can also be mapped to a black-and-white, gray-scale bar corresponding to the isotropic moiré (Fig. S1 bottom row), which can be used to explain the defect period and tilting angle.

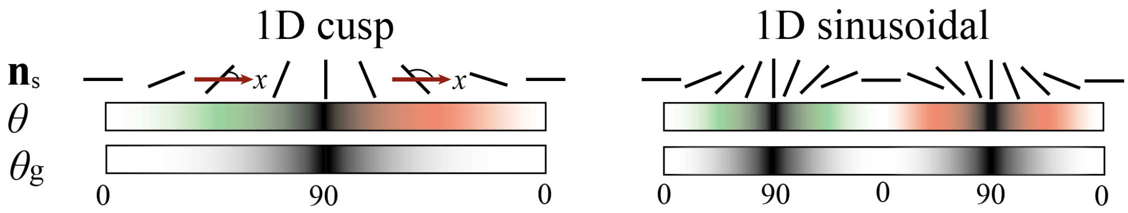

Figure S1: **LC surface anchoring pattern and the corresponding 1D geometric black-and-white gratings pattern in one period.** Top row: spatially varying surface-preferred director $\mathbf{n}_s$ (top), a colored bar corresponding to the surface-preferred orientation angle θ (middle), and the corresponding black-and-white gratings denoted by θ_g (bottom) along the x-direction. Left column: 1D cusp-like splay-bend pattern in a period L. Right column: 1D sinusoidal splay-bend pattern in a period L.

1.2 Theoretical prediction of defects in the thin-film limit

Here we apply a simple theory of disclination positions to our nematic moiré patterns. According to the theory, disclinations will appear where the surface-preferred (anchoring) orientations in the top and bottom surfaces have an angle difference of $\pi/2$ with twist reversal [1, 2, 3]. For the nematic moirés we consider in this manuscript, the anchoring director $\mathbf{n}_s$ is a 2D vector within the xy plane, and therefore can be described by its in-plane orientation angle θ, i.e., $\mathbf{n}_s = [\cos\theta, \sin\theta]$. To demonstrate the validity of this theory, in the following we consider the 1D cusp-like splay-bend pattern. We introduce θ^b and θ^t to denote the surface-preferred angle for the bottom and top substrate, respectively. The director angle at position $\mathbf{r} = [x, y]$ in the

bottom substrate is written as

$$\theta^b(x,y) = \theta^b(0,y) + \frac{\pi}{L}\mathbf{r}\cdot\mathbf{e}_x = \theta^b(0,y) + \pi x/L,$$

where L is the periodicity of the anchoring pattern, $\mathbf{e}_x = [1,0]$ is a unit vector pointing to the $+x$ direction, and $\theta^b(0,y)$ is a constant for the cusp-like pattern. Without loss of generality, we choose $\theta^b(0,y) \equiv 0$ in what follows. With the bottom substrate fixed, we consider a rotation operation over the top substrate by an angle Ψ with respect to the origin $[0,0]$ as the rotation center (see Ψ in Fig. 2A). Therefore,

$$\theta^t(x,y) = \frac{\pi}{L}\mathbf{R}^{-1}\cdot\mathbf{r}\cdot\mathbf{e}_x + \Psi, \tag{S1}$$

where $\mathbf{R}$ is the rotation matrix:

$$\mathbf{R} = \begin{bmatrix}\cos\Psi & -\sin\Psi \\ \sin\Psi & \cos\Psi\end{bmatrix}. \tag{S2}$$

Inserting the above into Eq. S1, we have

$$\theta^t(x,y) = \pi\left(x\cos\Psi + y\sin\Psi\right)/L + \Psi.$$

Thus, the angle difference $\Delta\theta$ is

$$\Delta\theta \equiv \theta^t(x,y) - \theta^b(x,y) = \frac{\pi}{L}\left(x\cos\Psi + y\sin\Psi - x\right) + \Psi.$$

We plot contour lines corresponding to $\theta^t(x,y) - \theta^b(x,y) = \frac{m\pi}{2}$, with $m = \pm1, \pm3, ...$ in Fig. S2A. These lines exactly match the disclinations found in the thin-cell simulations (Fig. S2B) and experiments (Fig. 1F) of the cusp-like pattern.

To view the periodicity of the angle difference, according to the nematic symmetry ($\mathbf{n} = -\mathbf{n}$), the angle difference $\Delta\theta$ can be casted into $[0, \pi/2]$ by the following:

$$\Delta\tilde{\theta} = \begin{cases}\arccos(\mathbf{n}_s^b\cdot\mathbf{n}_s^t), & \arccos(\mathbf{n}_s^b\cdot\mathbf{n}_s^t) \le \pi/2 \\ \pi - \arccos(\mathbf{n}_s^b\cdot\mathbf{n}_s^t), & \arccos(\mathbf{n}_s^b\cdot\mathbf{n}_s^t) > \pi/2\end{cases}$$

Re-scaled $\Delta\tilde{\theta}$ is shown in Fig. S2C.

1.3 Understanding the defect state diagram

The defect state diagram in Fig. 1F uses simulation to vary the gap-to-pattern ratio H/L and the rotation angle Ψ. It can be understood by considering the relative free energies of the three states.

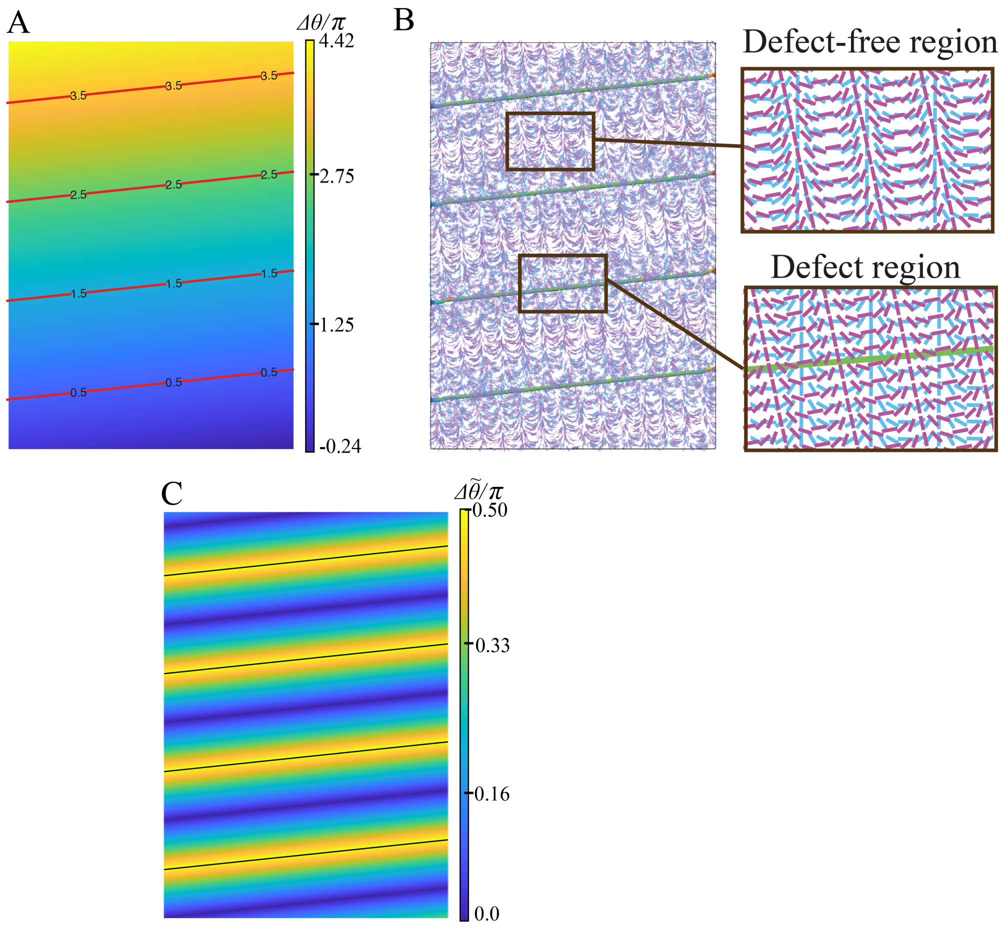

Figure S2: **1D cusp-like splay-bend pattern defect prediction and its simulation verification**. (A) $\Delta\theta = \theta^t - \theta^b = \frac{m}{2}\pi$ (contour lines for $\frac{m}{2}\pi$, with $m = \pm 1$, ± 3 ,....) and (B) corresponding simulation result of $H/L = 0.2$ (thin cell), purple for top substrate patterned director and blue for bottom substrate patterned director. Enlarged schematic views give a defect free region ($\Delta\tilde{\theta}$ close to 0) and a defect region ($\Delta\tilde{\theta}$ close to $\pi/2$). (C) Rescaled $\Delta\tilde{\theta} \in [0, \pi/2]$. Contour lines are for $\Delta\tilde{\theta} = \pi/2$. Source data are provided as a Source Data file.

When the cell gap is narrow (small H/L), the surface anchoring effect becomes important, which strongly frustrates the bulk nematic. The bulk nematic field represented by the orientation angle $\theta(x, y, z)$ adopts the surface-preferred orientations (θ^b or θ^t). The anchoring conflict between the two surfaces will cause a transition from $\theta(x, y, z) \approx \theta^b(x, y)$ near the bottom surface to $\theta(x, y, z) \approx \theta^t(x, y)$ near the top surface (Fig. S4A). Therefore, in the midplane of the cell, pure-twist-winding disclinations will appear where the angle difference of the two anchoring patterns becomes the maximum value, $\pi/2$. When the two substrates are away from each other (large H/L), the surface patterning effect becomes weak, and the bulk nematic dominates. Consequently, the system minimizes its elastic energy by aligning the nematic uniformly in the bulk and forming defects near the substrates to accommodate the anchoring conditions. The simple disclination-position theory indicates that line defects in the W-state should also appear when the difference between the surface-preferred angle and the bulk nematic orientation angle reaches the maximum value of $\pi/2$ (Fig. S4C). Because the bulk nematic field is uniform, the separation distance between neighboring line defects should coincide with the pattern periodicity L. This corollary is confirmed in both experiments and simulations (Fig. 1E, K, L).

At medium cell gaps, a delicate competition between the surface patterning and the bulk nematic leads to a complex defect structure, i.e., the C-state defect, the morphology of which is also intermediate between the parallel straight lines in the bulk for the S-state and the crossing lines near the two surfaces for the W-state (Fig. S4B). Similar to the W-state (Fig. S4C), the C-state forms a locally uniform director field in the midplane, and the defects are repelled to the two substrates (Fig. S4B). Note that the simple disclination-position theory cannot be used to predict the defect structure for medium cell gaps.

The rotation operation can also modify the disclinations in terms of their types and structural details (Fig. 1F, 2B). Similar to the effect of reducing the cell gap, increasing the rotation angle Ψ will induce more twist distortions in the system. To minimize the elastic energy cost, the system will more likely form pure-twist disclinations in the bulk for larger Ψ. Therefore, as Ψ increases, the system will favor the S-state against the C-state for thin cells and favor the W-state over the C-state for medium and thick cells (Fig. 1F, 2B, Movies S4, S5). The simulated polarizing optical microscopy (POM) images during rotation are well confirmed by the experiments, serving as an additional validation of the simulated 3D director field in the nematic moiré system (Fig. 2B). As Ψ increases, the dark grains are tuned from slender shapes to more round shapes (Fig. 2B), a feature similar to the isotropic moirés.

1.4 The details of the nematic structure

To further understand the structures of different types of defects, we introduce two angles to characterize the local profiles of line disclinations. The schematics of different local profiles of line disclinations are given in Fig. S3. The details of the S-, C- and W-state defect structures are shown in Fig. S4. Local defect structure is colored with a twist angle $\beta \in [0, \pi]$ and a "phase shift" angle $\alpha \in [0, 2\pi)$ [4, 5] (Fig. 1C–E, Fig. 2B, Fig. S4A and B). The local profile schematics are described in Fig. S3 and Topological analysis of disclination lines in Materials

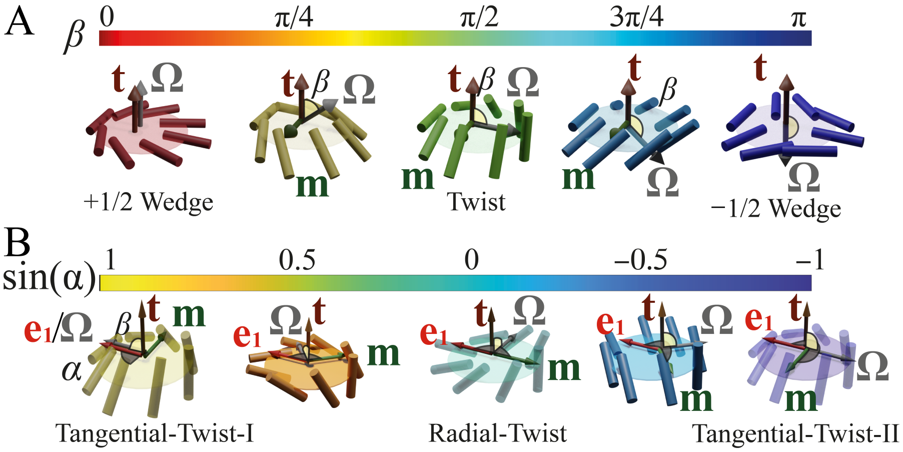

Figure S3: **Three-dimensional local profiles of disclination lines.** (A) a local $+1/2$ wedge winding continuously transforms into a $-1/2$ wedge through an intermediate twist winding. $\mathbf{t}$ is the tangent vector along the disclination curve. $\mathbf{\Omega}$ the unit rotation vector normal to the local director profile. M is normal to both $\mathbf{t}$ and $\mathbf{\Omega}$. (B) In a pure-twist disclination line, Tangential-Twist-I continuously transforms into Tangential-Twist-II through a Radial-Twist. Vector $\mathbf{e}_1$ living on the plane normal to $\mathbf{t}$ started from the defect core, see Topological analysis of disclination lines in Materials and Methods.

and Methods. Pure-twist and wedge winding correspond to $\beta = \pi/2$ and $\beta \neq \pi/2$, respectively. A pure-twist winding can be further characterized by an angle α, which can distinguish between two types of tangential twists ($\alpha = 0, 3\pi/2$) and two types of radial twists ($\alpha = 0, \pi$).

For the S-state, its winding exhibits a constant $\beta = \pi/2$, implying a uniform pure-twist profile (Fig. S4A). For the C-state, β varies within $[0, \pi]$. Near the two substrates, $\beta \approx \pi/2$, showing a pure-twist profile. When the disclination curve passes through the midplane of the cell, its local winding is of the wedge types, with β approaching 0 or π (Fig. S4B). The C-state presented in Fig. S4B is right-handed. If we rotate the top substrate clockwise, we see a left-handed helical structure (Fig. S5).

For the W-state, the defect structure is also of pure-twist type. To better distinguish the two groups of defects (one near the top substrate and the other near the bottom substrate) in the W-state, we use z/H to color the defects. From the middle plane director, we can observe that since defects are near the two substrates, the director is almost uniform in the mid plane (Fig. S4C). The simple theory that disclinations appear where top and bottom directors have an angle difference of $\pi/2$ with twist reversal is also valid here. Mid plane director in Fig. S4C is roughly along the x axis, so defects near the top and bottom surfaces are formed where the director on the patterns is along the y axis (see top plane and bottom plane in Fig. S4C).

A further topological characterization for the pure-twist defect type using the so-called "phase shift" angle α is performed over the S-state (Fig. S7) [5]. The periodic variation of α of the line defects in the S-state suggests that the local twist profile continuously varies between tangential-twist-I, radial-twist, and tangential-twist-II winding, albeit their simple geometry.

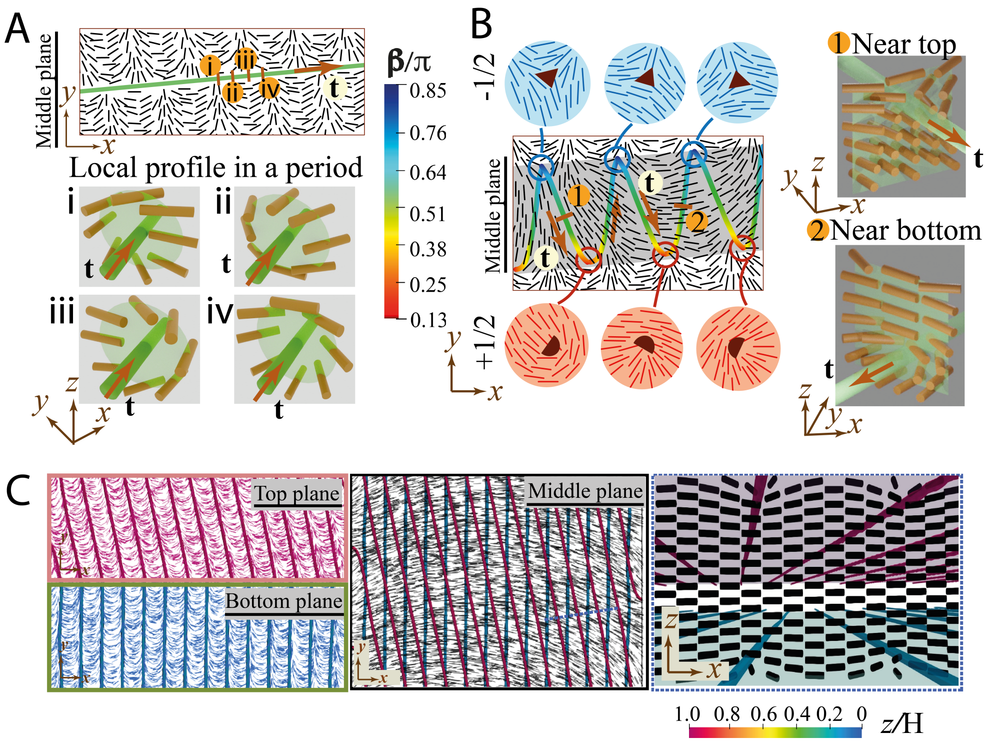

Figure S4: **The 3D view and director details of the S-, C- and W-state.** (A) The line defect in the S-state has constant pure-twist profile. (B) The defect in the C-state is a helical-like, 3D curve. (C) The W-state has two groups of defects. The group in pink is close to the top surface and the other group in blue is near the bottom.

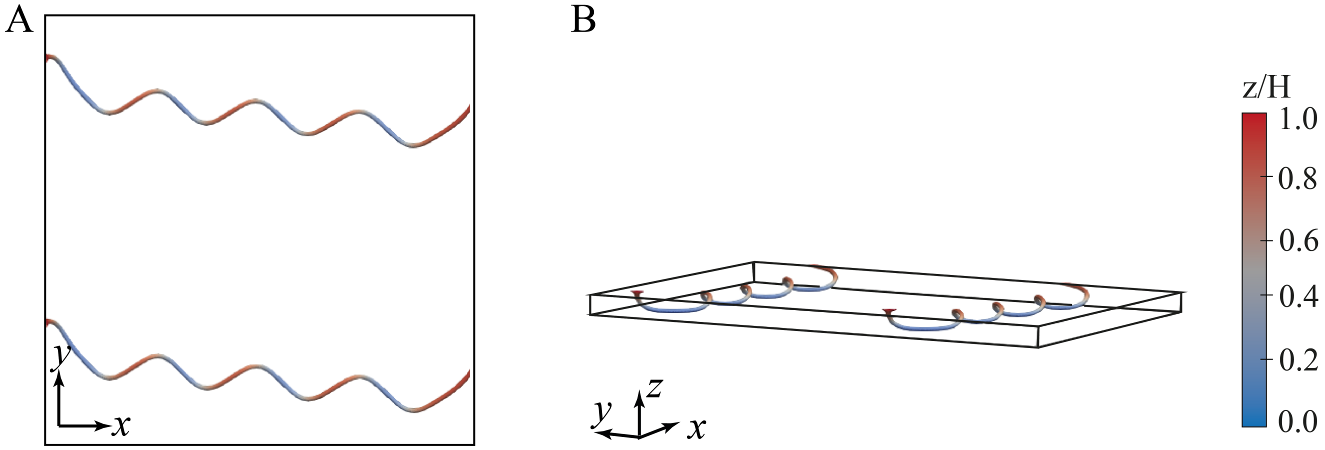

Figure S5: **Left-handed helical structure found in the cusp pattern by a clockwise rotation.** (A) $x - y$ view. (B) Angled view.

This contrasts with the line defect with uniform α in a similar system in which the top substrate adopts a uniform anchoring condition [6]. However, for the two groups of parallel defect lines in the W-state, the local director field along the curve tangent is nearly constant (Fig. S4C, top plane and bottom plane). Within the same group, all the parallel line defects share the same constant angle α.

Besides defect structures, simulations and experiments also agree on the POM images (the Jones matrix approach) for the three defect structures (Fig. S6). This optical pattern comprises parallel arrays of grain-like spots between neighboring defect curves. The periodicity of the grain array is roughly twice as the lattice constant L. These optical features are different from the isotropic moiré pattern formed from cosinusoidal gratings (Fig. S6 bottom).

Notably, the Jones-matrix approach is good for thin samples (Fig. S6A, B), while for thicker ones where focusing, ray deflection, and oblique rays become relevant, simulated images deviate from experiments (Fig. S6C). Adopting an approach by Poy [8], a better simulated optical image for the thick cell case is given in Fig. S38.

1.5 Periodicity and tilting angle of 1D geometric moiré and 1D nematic moiré

The essence of the (isotropic) geometric moiré is that the emergent overlapping pattern can be represented by its frequency vector, which is a linear combination of the frequency vectors of the two individual patterns. In what follows, we first elaborate the derivation of different moiré frequencies [7], then we measure the emerging periodicity in the nematic moiré for the S-, C-, and W-state, and resort to geometric moiré fringes for explanations.

1.5.1 $(1, -1)$-moiré

We denote the frequency vectors of two gratings by $\mathbf{f}_1$ and $\mathbf{f}_2$, respectively. Their frequencies and orientation angles (with respect to the horizontal axis) are represented by f_i and Ψ_i, respectively, with $i = 1, 2$. The two vectors can therefore be written as $\mathbf{f}_i = (f_i \cos\Psi_i, f_i \sin\Psi_i)$ in the frequency space. For the $(1, -1)$-moiré, the emergent frequency is $\mathbf{f} = \mathbf{f}_1 - \mathbf{f}_2$ (Fig. S8). The two components of the vectorial sum $\mathbf{f} = (u, v)$ can be written as

$$u = f_1 \cos\Psi_1 - f_2 \cos\Psi_2 \tag{S3}$$

and

$$v = f_1 \sin\Psi_1 - f_2 \sin\Psi_2. \tag{S4}$$

Thus, the superposed frequency is

$$\begin{aligned} f &= \sqrt{u^2 + v^2} \\ &= \sqrt{f_1^2 + f_2^2 - 2 f_1 f_2 \cos(\Psi_2 - \Psi_1)}. \end{aligned}$$

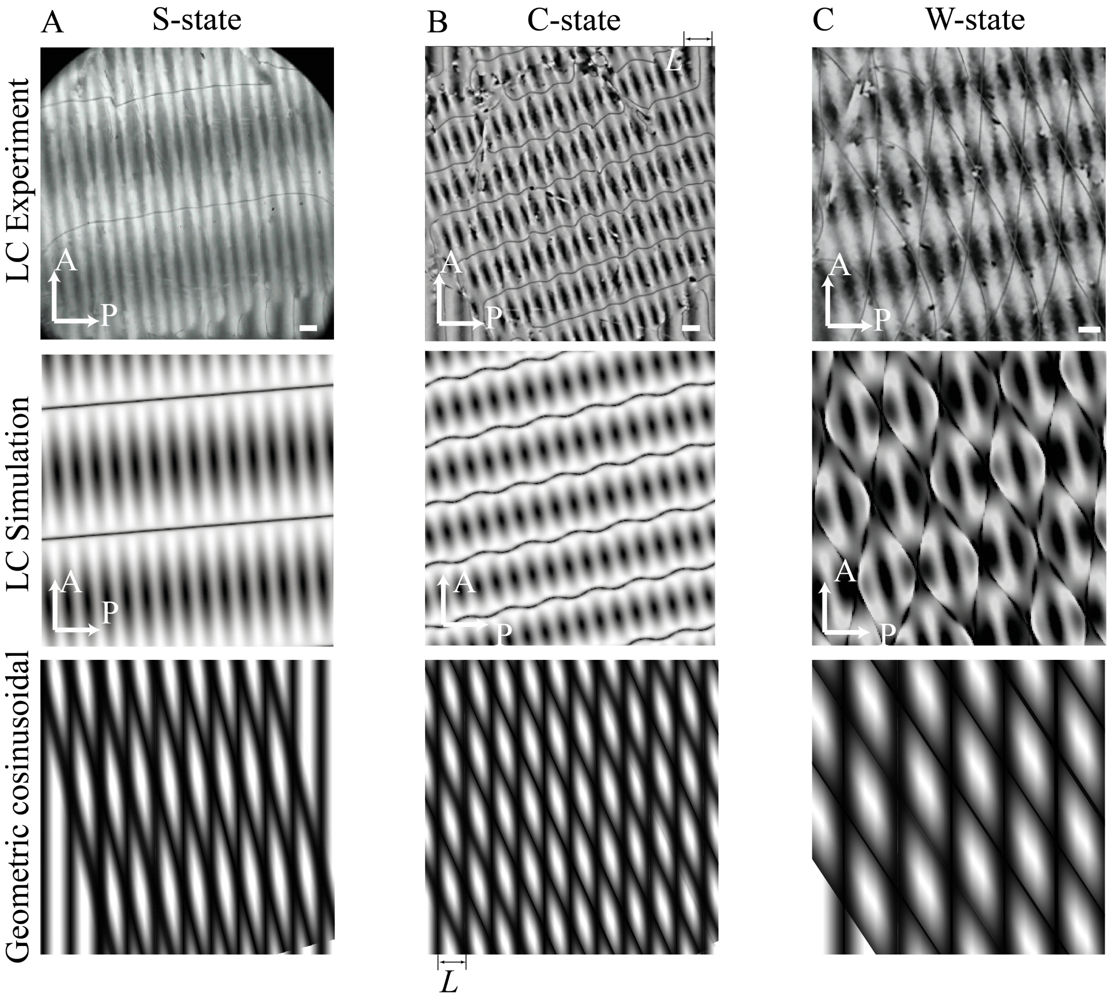

Figure S6: **The comparison of the optical pattern generated in the experiment and simulation (using 1D cusp-like splay-bend pattern), and the corresponding overlaid isotropic cosinusoidal patterns.** In each column, moiré period T and lattice constant L of the experimental POM image, the simulation POM image and geometric cosinusoidal gratings are kept identical. The constant L is denoted in (B) (top). Scale bar: $50\ \mu$m.

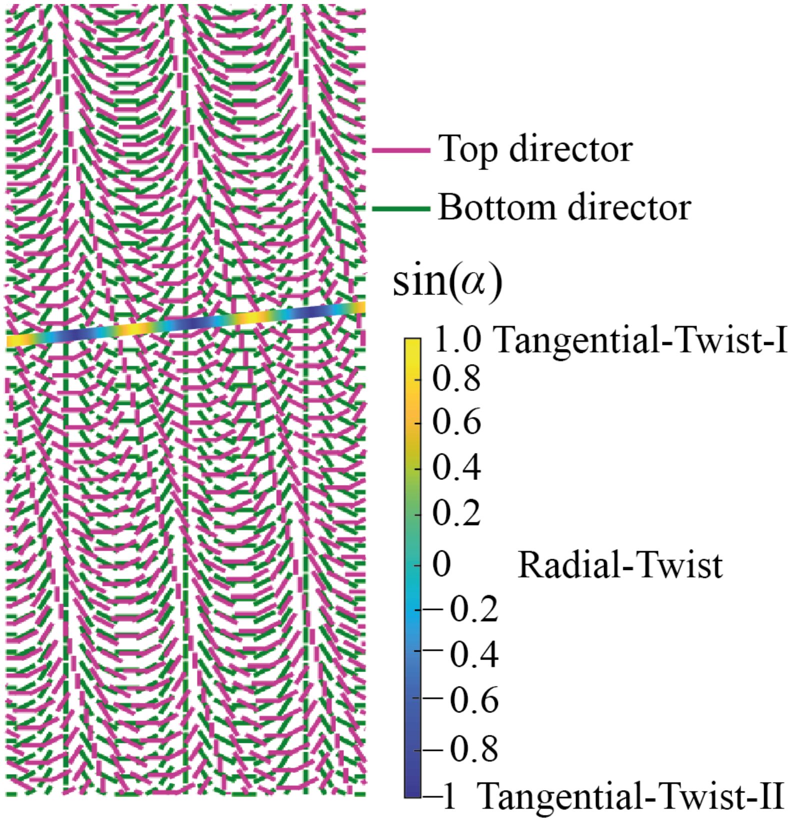

Figure S7: α **angle of the line defects in the S-state**. $\sin\alpha$ varies from -1 to 1 periodically.

Let $\Psi = \Psi_2 - \Psi_1$ be the relative twist angle, and we can obtain the superposed period T as

$$\frac{1}{T^2} = \frac{1}{T_1^2} + \frac{1}{T_2^2} - \frac{2\cos\Psi}{T_1 T_2}.$$

In our pattern, the two gratings have the same periodicity, $L = T_1 = T_2$, therefore,

$$T = \frac{L}{\sqrt{2(1-\cos\Psi)}}. \tag{S5}$$

The tilting angle ω in the moiré is

$$\omega = \arctan\frac{v}{u}, \tag{S6}$$

By plugging Eq. S3 and Eq. S4 into Eq. S6, we have

$$\omega = \arctan\frac{\sin\Psi}{\cos\Psi - 1} = \frac{\Psi}{2}. \tag{S7}$$

The deduced T and ω can also be applied to $(-1,+1)$-moiré.

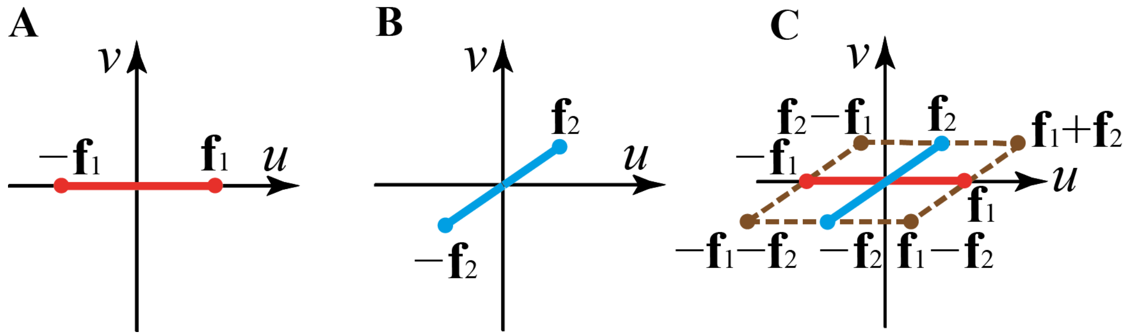

Figure S8: **Frequency convolution**. (A) and (B) are two original grating spectra, and (C) is their convolution showing $(\pm 1, \mp 1)$-moiré and $(\pm 1, \pm 1)$-moiré.

1.5.2 $(1, 1)$-moiré

For $(1, 1)$-moiré, the summed vectorial frequency is $\mathbf{f}' = \mathbf{f}_1 + \mathbf{f}_2$ (Fig. S8). The two components of the vector $\mathbf{f}' = (u', v')$ in frequency space can be written as

$$u' = f_1 \cos \Psi_1 + f_2 \cos \Psi_2$$

and

$$v' = f_1 \sin \Psi_1 + f_2 \sin \Psi_2.$$

The superposed frequency f' is

$$\begin{aligned} f' &= \sqrt{u'^2 + v'^2} \\ &= \sqrt{f_1^2 + f_2^2 + 2 f_1 f_2 \cos(\Psi_2 - \Psi_1)}, \end{aligned}$$

which also applies for $(-1, -1)$-moiré. Let $\Psi = \Psi_2 - \Psi_1$, we obtain the superposed period T'

$$\frac{1}{T'^2} = \frac{1}{T_1^2} + \frac{1}{T_2^2} + \frac{2\cos\Psi}{T_1 T_2}.$$

In our pattern, two gratings have the same periodicity, $L = T_1 = T_2$, therefore,

$$T' = \frac{L}{\sqrt{2(1 + \cos\Psi)}}.$$

From Fig. S9, vector $\mathbf{f}_1 + \mathbf{f}_2$ and $\mathbf{f}_2 - \mathbf{f}_1$ are perpendicular to each other, the tilting angle difference should be $\pi/2$, so

$$\omega' = \omega + \pi/2.$$

A comparison of T/L and T'/L is depicted in Fig. S9. As Ψ goes from 0 to $\pi/2$, T gradually decreases while T' slowly increases. Note that $\Psi = 0$ is a singular point.

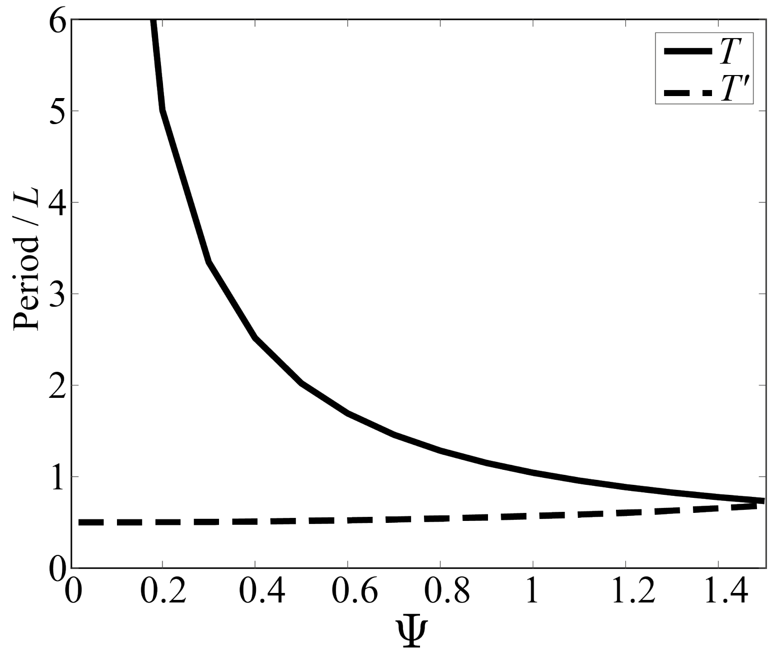

Figure S9: **Normalized period T/L and T'/L versus twist angle Ψ.** Source data are provided as a Source Data file.

1.5.3 T, T' and ω in the nematic moiré

To understand the geometry of the three defect structures more quantitatively, we proceed to measure the spacing T between the neighboring parallel disclinations (Fig. 2B) and their orientation angle ω (with respect to the x-axis, see Fig. 2B) as functions of Ψ for the S- and C-states. For the W-state, we first introduce a pseudo line connecting disclination crossings of neighboring pairs of line defects (dashed lines in Fig. 2E inset); T and ω then denote the spacing between these parallel pseudo lines and their orientation angles, respectively. As Ψ increases, the disclinations become closer to each other, and their orientations further rotate (Fig. 2B, Movie S6). A larger Ψ gives rise to more frequent variations in the anchoring angle mismatch $\Delta\theta$ in space, and the disclinations therefore appear denser in the system. Both T and ω can be understood by the geometric moiré pattern. The theoretical values Eq. S5 and Eq. S7 for the first-order low frequency $(\pm 1, \mp 1)$-moiré in the geometric moiré pattern (Fig. S9) are quantitatively matched by those measured from all three defect states in both simulations and experiments (Fig. 2D, E). For the C-state, there are two emerging geometric parameters associated with the helical-like defects, namely, the period of the helix T^* and its helical diameter projected onto the xy plane A_{xy} (Fig. 2B). For the W-state, T^* is defined as the spacing between adjacent defect crossings on the same pseudo line. It turns out that T^* in both states can be understood by the periodicity T' for the $(\pm 1, \pm 1)$-mode in the geometric moiré pattern (Fig. S8, S9), as $T^*/T' = 2$ in both states (Fig. 2F, G). The amplitude of the defects A_{xy} in the C-state increases as Ψ decreases (Fig. 2F, Movie S6) or H/L increases (Fig. 2G, Movie S1). Upon the transition from the C-state to

the W-state, A_{xy} approaches T as the neighboring wavy-like defects intersect and start to form web-like defects (Fig. 2F, G, Movie S1). The above remarkable results show that the geometric details of the emerging disclinations, regardless of their types, are fundamentally dictated by the geometry of the moiré pattern, and different length scales emerged from the defect structures stem from different moiré modes, the high-frequency ones of which are usually difficult to see in isotropic moiré patterns. Therefore, our nematic moiré pattern provides a simple method to tune the topology and geometry (shapes, periodicities, orientations, etc.) of the disclinations using the geometry of moiré patterns.

2 1D sinusoidal splay-bend pattern

2.1 Defect structure prediction in thin-film limit

Following the same procedure in the theoretical prediction of the disclinations in the cusp-like pattern, we first express the anchoring orientation angle θ^b at location $\mathbf{r} = [x, y]$ on the bottom substrate as

$$\theta^b(x,y) = \theta^b(0,y) + \frac{\pi}{2}\sin(\frac{2\pi}{L}\mathbf{r}\cdot\mathbf{e}_x) = \theta^b(0,y) + \frac{\pi}{2}\sin(2\pi x/L).$$

Next, the top substrate is rotated by Ψ with respect to the origin $[0, 0]$. By dropping the constant $\theta^b(0, y)$, we have

$$\theta^t(x,y) = \frac{\pi}{2}\sin(\frac{2\pi}{L}\mathbf{R}^{-1}\cdot\mathbf{r}\cdot\mathbf{e}_x) + \Psi. \quad \text{(S8)}$$

By inserting Eq. S2 for $\mathbf{R}^{-1}$ into Eq. S8, we have

$$\theta^t(x,y) = \frac{\pi}{2}\sin(2\pi\left(x\cos\Psi + y\sin\Psi\right)/L) + \Psi. \quad \text{(S9)}$$

Thus, the director angle difference is

$$\Delta\theta = \theta^t(x,y) - \theta^b(x,y) = \frac{\pi}{2}[\sin(2\pi\left(x\cos\Psi + y\sin\Psi\right)/L) - \sin(2\pi x/L)] + \Psi.$$

The theory predicts that defects will appear in where $\Delta\theta = \frac{m\pi}{2}$, for $m = \pm 1, \pm 3, ...$ in Fig. S10A [1, 2, 3]. Thin-film simulation results in Fig. S10B show that defects are exactly generated in where the theory predicts. Fig. S10A also reveals $\Delta\theta$ varies periodically and forms defect loops of two sizes. Large red contour loops exhibit with positive $\Delta\theta$, which decreases from inside out of the loop. Whereas, blue contour loops show negative $\Delta\theta$, which increase from inside out of the loop (Fig. S10A and Fig.3). Note that when $\Psi = 0$, $\Delta\theta = 0$ and defects are not generated.

Again, this prediction can be only applied to thin cells, in which surface anchoring effect dominates. As shown in Fig. S11A and B (left), thin cell simulation ($H/L = 0.08$) has a perfect match with the prediction in Fig. S11A, while the group of small loops (in blue) annihilates when $H/L \geq 0.1$. Experimental results of a medium cell in Fig. S11C agrees with Fig. S11B ($H/L = 0.1$).

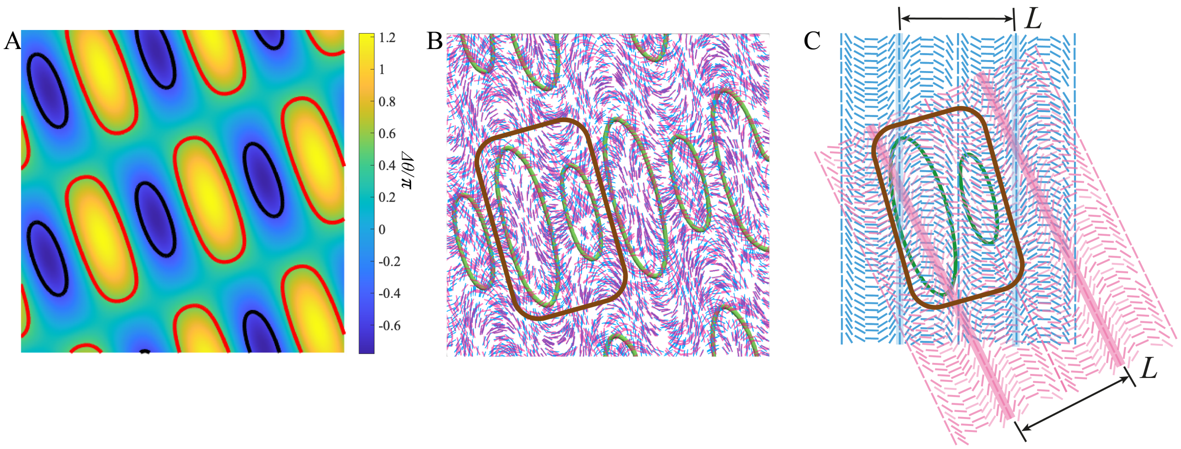

Figure S10: **1D sinusoidal cell defect prediction and its simulation verification**. (A) Theoretical prediction: $\Delta\theta = \theta^t - \theta^b$ of 1D sinusoidal pattern. Red and blue contour lines are for $\Delta\theta = \pi/2$ and $\Delta\theta = -\pi/2$, respectively. Source data are provided as a Source Data file. (B) Corresponding simulation result in the $H/L = 0.1$ cell, pink for top substrate pattern and blue for bottom substrate pattern. The schematic for the enlarged view of the framed region in (B) is (C).

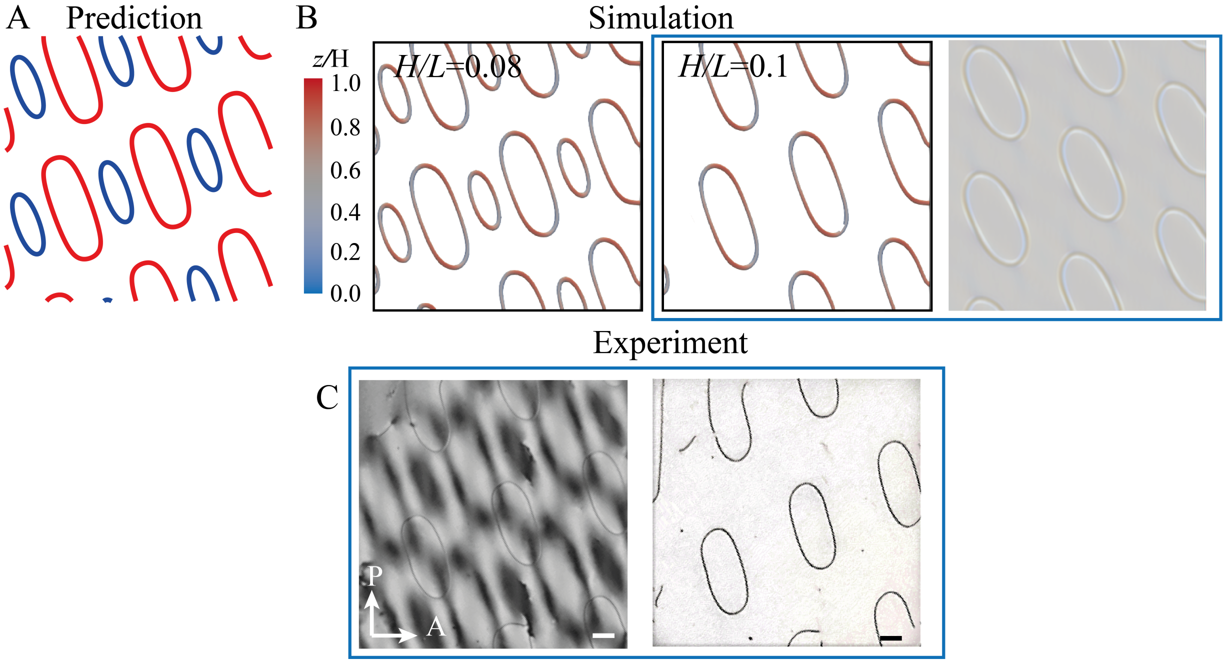

Figure S11: **Defect prediction, simulation and experiment at $\Psi = 40°$.** (A) The theoretic prediction with larger loops in red and smaller loops in blue. (B) Simulation of two cell thicknesses, a thinner cell of $H/L = 0.008$ (agree with the prediction) and a thicker cell of $H/L = 0.1$. The simulated optical image is generated using the open-source code by G. Poy [8]. (C) Medium cell results in the experiments agree with $H/L = 0.1$ simulation. POM image (left) and bright field image (right). Scale bar: $50\ \mu$m.

2.2 The details of the nematic structure

Movie S9 shows the evolution of loops with the change of the cell gap. With the imposed patterns at the top and bottom substrates, a position-dependent twist is induced in the bulk achiral nematic. For a thin cell, the surface anchoring effect is important and the twist-winding disclinations appear where the angle difference $\Delta\theta(x, y)$ is $\pi/2$. When the two substrates are away from each other (large H/L), the surface patterning effect becomes relatively weak and the bulk nematic dominates. Smaller loops are less stable and gradually self-annihilate. As the cell becomes even thicker, the system chooses to minimize its elastic free energy by aligning the nematic uniformly in the bulk and forming defects near the substrates to accommodate the surface anchoring pattern. The emergence of the W-state is observed.

The patterning effect is weakened as the cell gets thicker, as depicted in Fig. S12. Looking at the mid plane director ($z/H = 0.5$) (Fig. S12), we see the annihilation of smaller defect loops (Loop-I) when cell gap increases. Viewing the mid plane ($z/H = 0.5$) in Fig. S12B, Loop-I has two $-1/2$ wedge local profiles and Loop-II has two $+1/2$ wedge local profiles. As the cell gets thicker, Loop-II evolves into Loop-III (Fig. S12C, D), and eventually a web-like defect structure is formed when $H/L = 0.33$ (Fig. S12E, F).With the local profile topology further characterized by angle β, Fig. S13 shows the three kinds of defect loops that appear in 1D sinusoidal pattern. The three kinds are all different from the typical neutral wedge-twist loop (Fig. S13D [4]). Note that there is no more than a π rotation from the top to the bottom patterned substrate, therefore the formation of knots is topologically forbidden. Disclination loops, like hedgehog point defects, carry an integer hedgehog charge [4, 9]:

$$d = \frac{1}{4\pi} \int_{\mathbb{S}^2} \mathrm{d}\theta \mathrm{d}\phi \mathbf{n} \cdot [\partial_\theta \mathbf{n} \times \partial_\phi \mathbf{n}].$$

The topological charge of the three kinds of loops is zero and they can all disappear or self-annihilate upon rotation or cell gap change.

2.3 Defect configurations at different rotation angle Ψ

In the 1D sinusoidal splay-bend pattern, defect periodicity T and T^*, as well as tilting angle ω in simulations and experiments can also be explained by the isotropic moiré. An example of $H/L = 0.1$ is shown in Fig. S14. Similar to the 1D cusp-like pattern, as Ψ increases, the projected diameters of the loop in the xy plane, A_1 and B_1, decrease. The fates of the two types of loop can be understood by the spatial distribution of $\Delta\theta$. As rotation angle Ψ increases, we expect that the anchoring angle difference $\Delta\theta$ also increases. This is consistent with the fact that the inside region of loop I corresponding to small $\Delta\theta$ will shrink and even self-annihilate at increasing Ψ (Fig. S14F). However, the transformation of loop II as a function of Ψ is more complicated, as is reflected by the non-monotonic dependence of its area on Ψ (Fig. S14H). At $\Psi = 90°$, the defect loops become multi-stable. In both simulations and experiments, loops can be either connected or isolated. By heating the sample to isotropic phase and then cooling it back to the nematic phase, different stable states are observed in the experiments (Fig. S15 C).

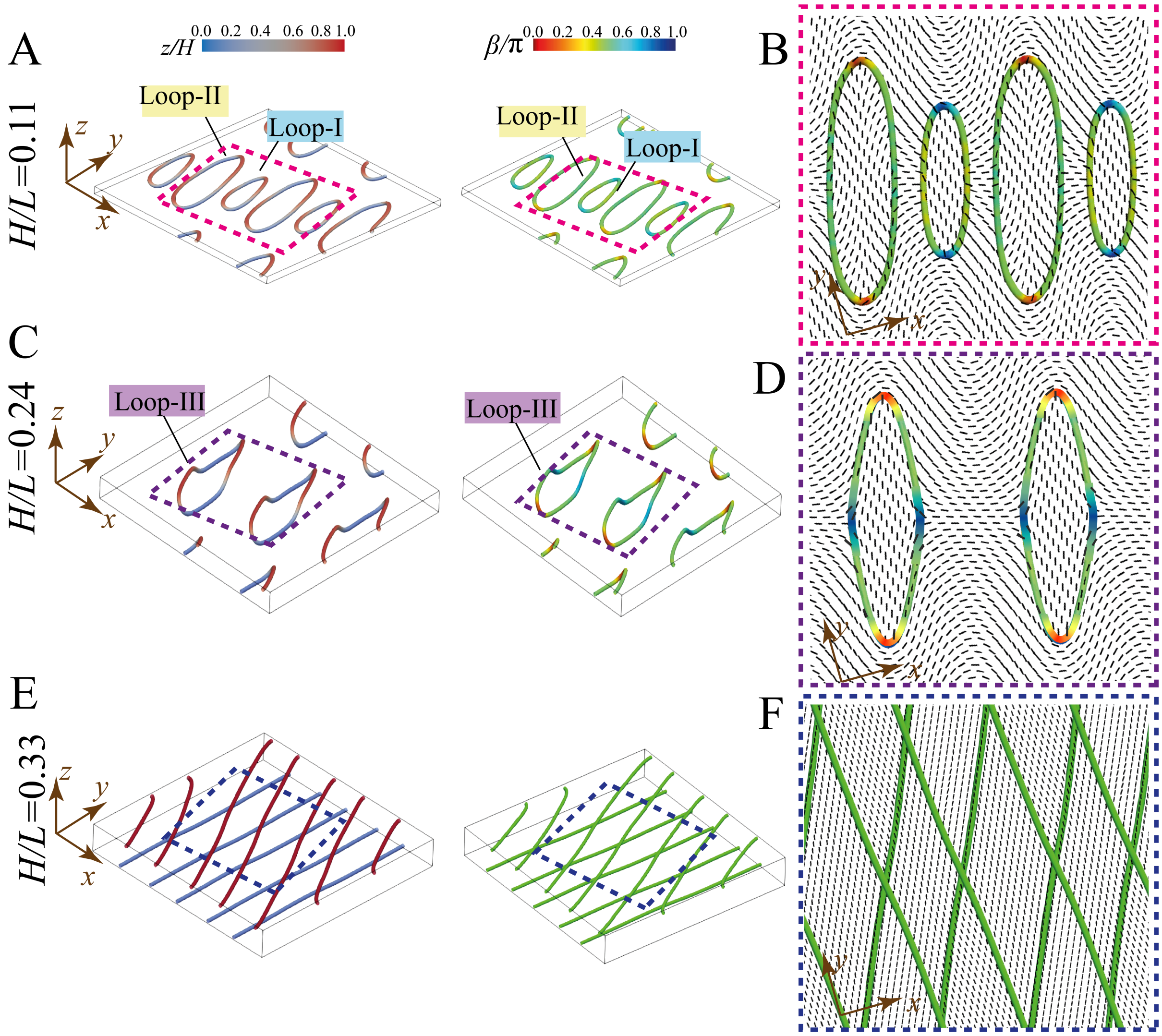

Figure S12: **Director field and defect configuration change with the cell thickness (at $\Psi = 30°$)** (A) 3D view of defects at $H/L = 0.11$, with Loop-I and Loop-II emerging. Defect structures are colored by z/H and β. (B) Planar view of defects and mid plane ($z/H = 0.5$) director around the four loops in A (pink frame). (C) 3D view of defects at $H/L = 0.24$. Loop-I has annihilated and Loop-II now evolves into Loop-III. (D) Planar view of defects and mid plane ($z/H = 0.5$) director around the two loops in (C) (purple frame). (E) 3D view at $H/L = 0.33$, defects are repelled near the two patterned surfaces. Defect structures are colored by z/H and β. (F) Planar view of defects and mid plane ($z/H = 0.5$) director of the framed region in (E). (B), (D) and (F) are colored by β.

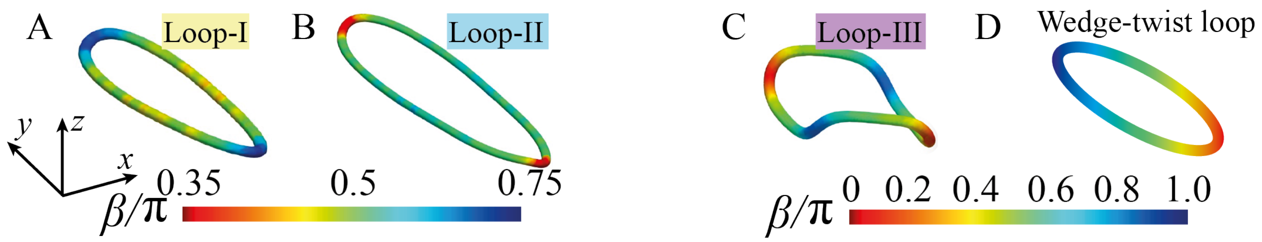

Figure S13: **Local profile of four topological loops characterized by β.** (A) Loop-I. (B) Loop-II. (C) Loop-III. (D) The typical wedge-twist loop [4].

As we increase the angle Ψ from 0, the defects first enter the simulation window around $\Psi = 6°$ as shown in Fig. S14A. Then we observe one array of closed defect loops at $\Psi = 12°$ (Fig. S14B) and multiple arrays of defect loops with a distance of T after $\Psi > 24°$ We also have characterized the aspect ratio change in the Supplement Information. We define parameters A_1, A_2, B_1, and B_2 to represent the shape change of the loops with Ψ, shown in Fig. S14A. And Loop-I annihilates at $\Psi = 58°$. Differently, in the theory (thin cell limit), Loop-I annihilates (becomes a point) at $\Psi = 80°$ since the anchoring effect from the patterns plays a more important role than the simulation with $H/L = 0.1$ (Fig. S14H). Loop-II has a larger projected area (in $x - y$ plane) and aspect ratio than Loop-I. While the theory over-estimates the defect loop sizes (Fig. Fig. S14H), it makes accurate prediction of their aspect ratios (Fig. S14I). As a result, the theory overestimates the self-annihilation point. Take Loop-I as an example: theory predicts $\Psi = 80°$, compared to $\Psi = 58°$ in the simulation (Fig. S14H).

3 2D defect lattice

3.1 Cell gap effect

Similar to the 1D pattern results, as we increase H/L, defects are gradually repelled from the bulk and appear near the two substrates. In the thin-cell limit (Fig. S16A), defect curves appear straighter, akin to the S-state of the 1D cusp-like pattern. Thick cell simulation shows a web-like defect akin to the W-state in the 1D cusp-like pattern (Fig. S16C).

3.2 Defect configurations at different rotation angles Ψ

Before rotation ($\Psi = 0°$), defects are all loop-like (Fig. S17). Fig. S18 shows the defect structure changing with Ψ. Green dashed lines are for the periodic unit cells (to illustrate the heterogeneous types of disclinations). Fig. S17 gives the configuration of quasi-loops in 3D, colored by the normalized z coordinate. In the planar view of the experiment, a quasi loop like defect appears as two dots due to the fact that the defects are split from the surfaces. Then we further characterize topological structures using angle β in simulations. As shown in Fig. S17C-

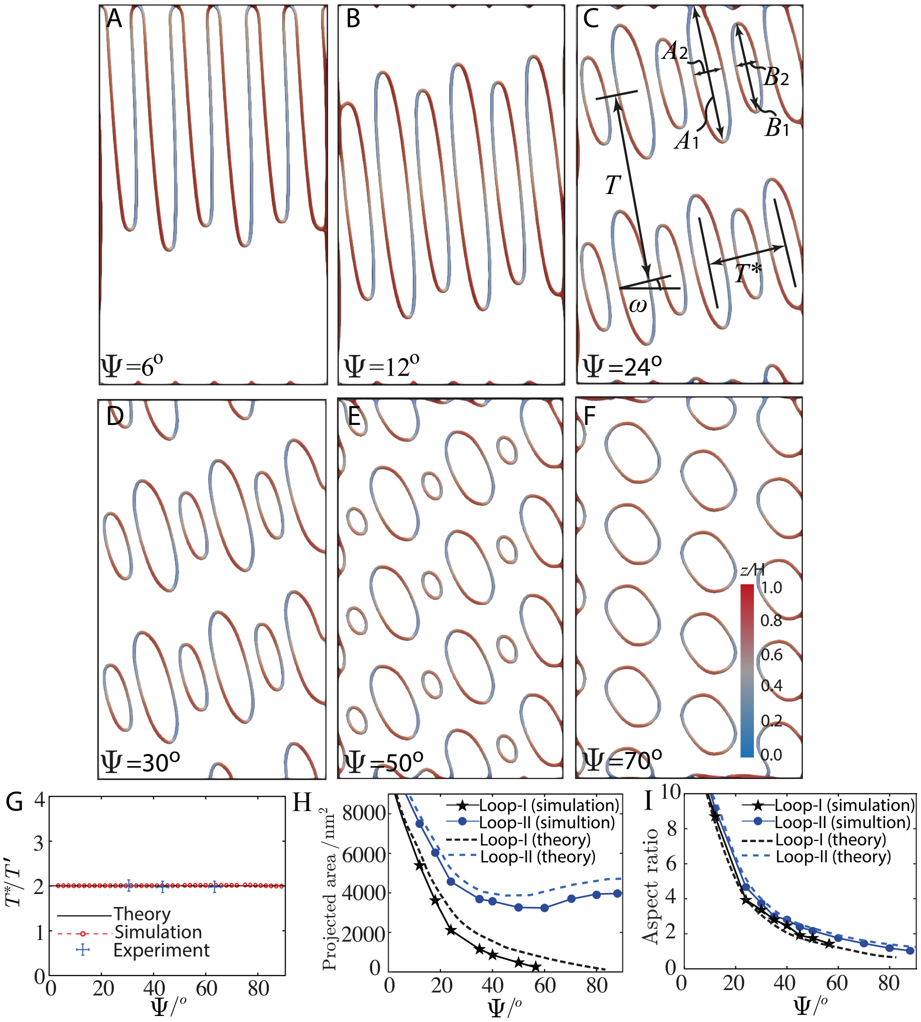

Figure S14: **Defect structure at different** Ψ $(H/L = 0.1)$**.** (A) $\Psi = 6°$. (B) $\Psi = 12°$. (C) $\Psi = 24°$. (D) $\Psi = 30°$. (E) $\Psi = 50°$. (F) $\Psi = 70°$. (G) The change of normalized period T^*/T' over rotation angle Ψ. (H) The projected area in the xy plane of Loop-I and Loop-II in simulations and theories. (I) The aspect ratio of Loop-I and Loop-II in simulations and theories. Source data are provided as a Source Data file.

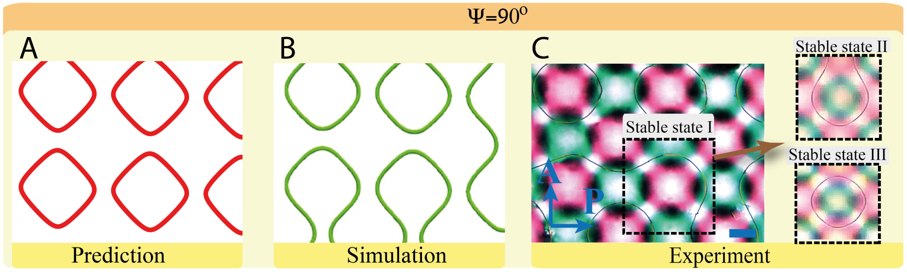

Figure S15: **Defect structures are multi-stable at** $\Psi = 90°$ $(H/L = 0.1)$**.**

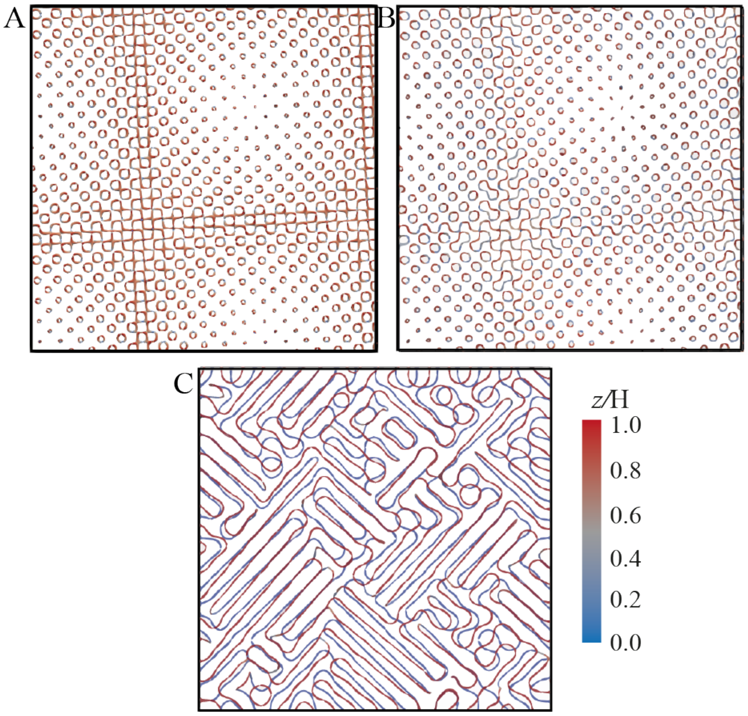

Figure S16: **Defect structures with different cell gaps (at $\Psi = 5°$)** (A) $H/L = 0.12$. (B) $H/L = 0.3$. (C) $H/L = 1.2$.

D, a quasi-loop that connects two $+1$ defect cores show $\beta \in [0, \pi/2]$: near the two surfaces, β approaches $\pi/2$ while $+1/2$ wedge profile appears in the bulk with $\beta \approx 0$. On the other hand, a quasi-loop that connects two -1 defect cores exhibit $\beta \in [\pi/2, \pi]$: near the two surfaces, β approaches $\pi/2$ while $-1/2$ wedge profile appears in the bulk with $\beta \approx \pi$. These two arc-like defects repel each other elastically and appear as two short lines when viewed from top (Fig. S17B).

3.3 The details of the nematic structure for $\Psi = 5°$

At a small rotation angle (e.g., $\Psi = 5°$), the dot screen moiré corresponding to the 2D nematic moiré appears as a super lattice of overlapping lattice points (Fig. 5B). This super lattice is a tilt square lattice with a lattice constant T and an orientation angle ω (Fig. 5B). In our nematic moiré pattern simulations, we observe a 2D periodic structure of disclinations, which coincide with the periodicity of the geometric moiré (Fig. 5G, H, Movie S11).

In the different regimes of the super lattice, different disclination structures are observed (Fig. 5D). In a unit period cell of the super lattice (dashed green grids in Fig. S18), there are three distinctive defect regions, the edge (Region I), the corner (Region II), the center (Region III) of the unit cell , presented in Fig. S19. Due to the local differences in the moiré patterns, the defect shapes are distinct in different regions. Near the center of each unit cell (green dashed lines in Fig. 5D and Fig. S18) of the nematic moiré, isolated loop-like defects emerge. In

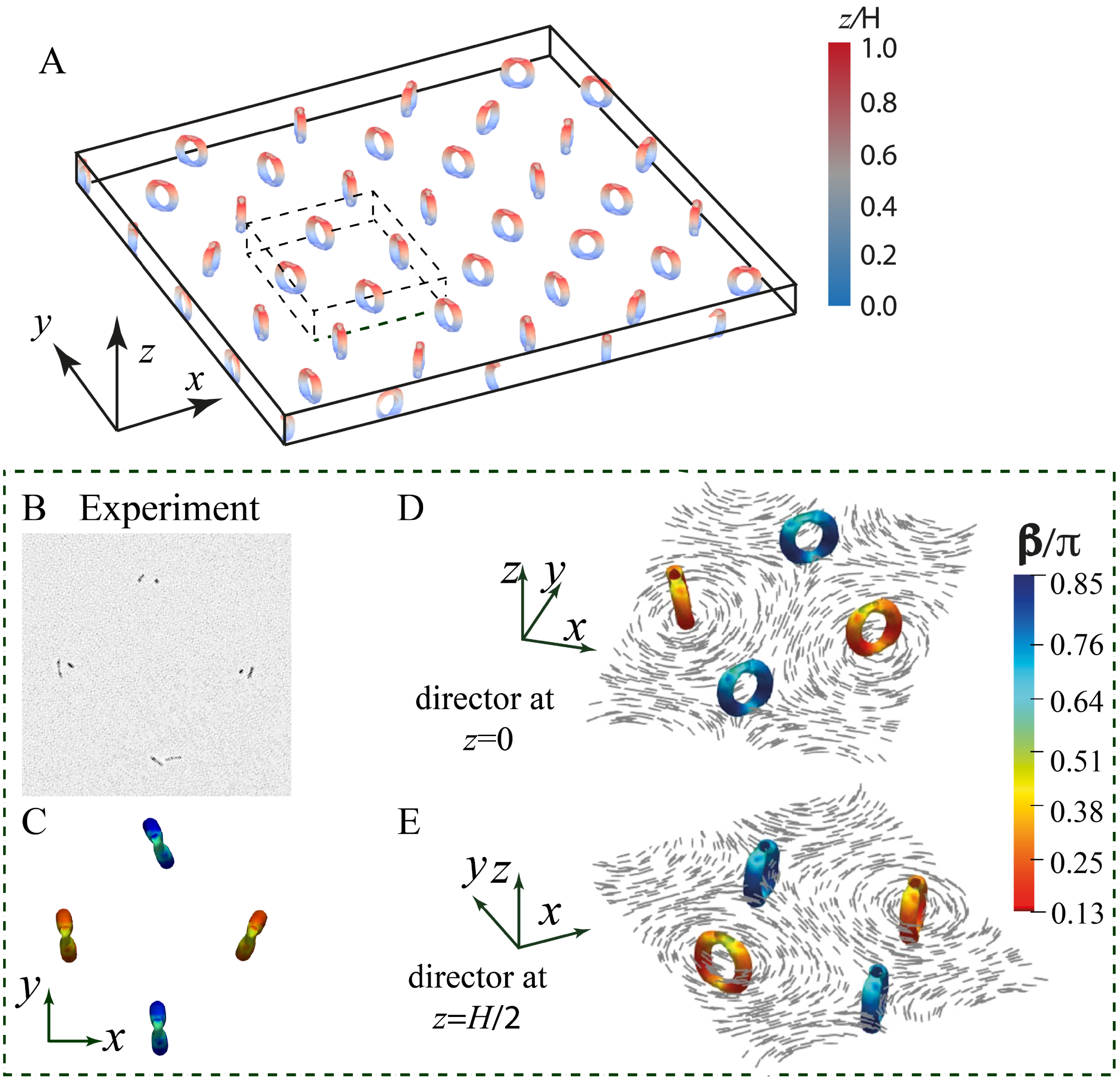

Figure S17: **Loop-like defect array at $\Psi = 0°$ in 2D defect pattern.** (A) Arrays of loop-like defects in the 3D view (simulation). (B) Experimental bright field snapshot corresponding to the boxed region in (A) ($x - y$ view). (C)-(E) are different views of simulation results characterized by angle β. (D) shows the director field at the bottom plane ($z = 0$) and (E) gives the director field in the mid-plane ($z = H/2$).

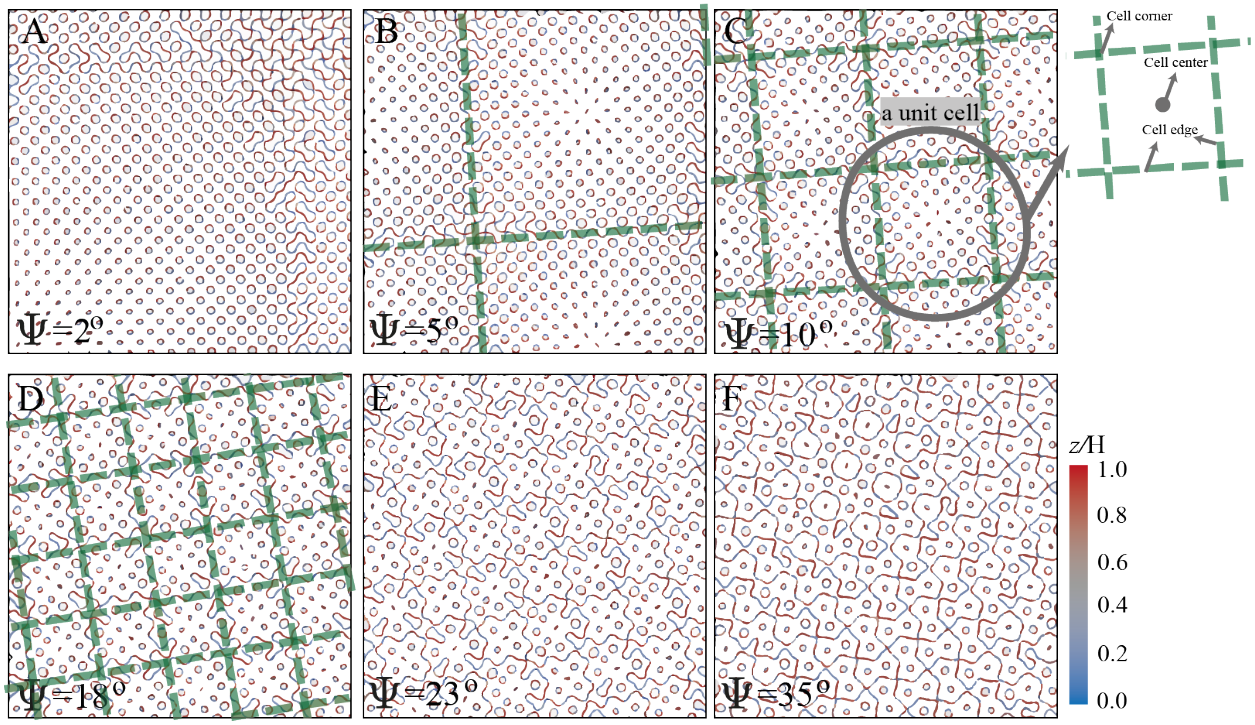

Figure S18: **Defect configurations at different Ψ.** Periodic uniform cells with size $T \times T$ are edged by dashed green lines.

region I, their sizes expand as they are further away from the center (Fig. 5D, E, Fig. S19), while the mismatch of the (x, y) coordinates of the pairing surface defects increases. Due to the anchoring mismatch, quasi-loops with $+1/2$ $(-1/2)$ wedge profile near the midplane are formed at the center of the unit cell (Fig. S19A inset) while the loops are elongated with β approaching $\pi/2$ as they are further away from the center (the color change of the loops in enlarged view in Fig. S19A). Therefore, there is a correlation between the loop size and the twist angle of its windings in the ± 1 pattern system. Near the four edges of the unit cell (Region II), the spatial correlation of the integer defects on the opposite surfaces is frustrated, leading to the formation of interconnected disclination lines (green dashed lines in Fig. 5D and Fig. S19). The above simulated disclination structures, confirmed in the experiment (Fig. 5E, F), persist up to $\Psi \approx 21°$.

3.4 2D geometric moiré theory

For a 2D geometric moiré, we consider two 2D square lattices of dot screen pattern, each of which comprises two orthogonally superposed 1D gratings. The resulting "impulse" (pattern) can be represented by a 4D index array, (k_1, k_2, k_3, k_4), with the first two indexes describing one (bottom) pattern and the last two representing the other (top) pattern [7]. The highest of the absolutes of the four indexes is the order of the impulse. Let the frequency magnitude of the two square lattices be f_1 and f_2. The vectorial sum $\mathbf{f}$ will be a linear combination of four vectors. By assuming one lattice aligned with the Cartesian coordinate and the other lattice rotated by an angle Ψ with respect to the first pattern, the two elements of $\mathbf{f} = (u, v)$ in the frequency space

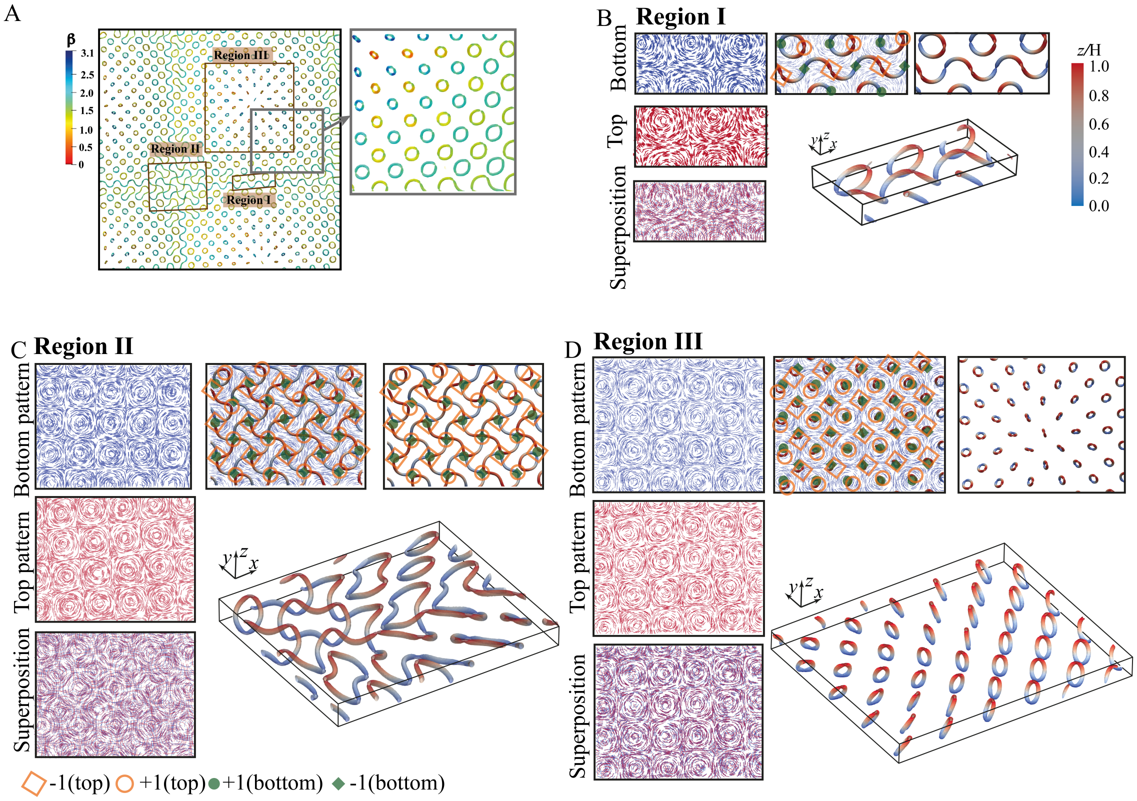

Figure S19: **Defect details at** $\Psi = 5°$**.** (A) Defect shape at $\Psi = 5°$. Three labelled regions and an enlarged region are framed. (B) Director field of the bottom pattern, the top pattern, the superposed anchoring pattern, and defect structure in planar view and 3D view of "Region I" (at periodic defect cell edge). (C) Director field of the bottom pattern, the top pattern, the superposed anchoring pattern, and defect structure in planar view and 3D view of "Region II" (at periodic defect cell corner). (D) Director field of the bottom pattern, the top pattern, the superposed anchoring pattern, and defect structure in planar view and 3D view of "Region III" (at periodic defect cell center). ± 1 defect cores on both substrates are noted.

can be written as

$$u_{k_1,k_2,k_3,k_4} = f_1k_1 + f_2\left(k_3\cos\Psi + k_4\cos(\pi/2+\Psi)\right),$$

and

$$v_{k_1,k_2,k_3,k_4} = f_1k_2 + f_2\left(k_3\sin\Psi + k_4\sin(\pi/2+\Psi)\right).$$

Hence, its frequency $f_{1,2,3,4}$ and period $T_{1,2,3,4}$ are

$$f_{k_1,k_2,k_3,k_4} = \sqrt{u^2_{k_1,k_2,k_3,k_4} + v^2_{k_1,k_2,k_3,k_4}},$$

and

$$T_{k_1,k_2,k_3,k_4} = 1/f_{k_1,k_2,k_3,k_4}.$$

In 2D gratings, the number of moirés within the visible circle is greater than that of the 1D case, but many of them are too weak to be visible. When $T_1 = T_2 = T_3 = T_4$ as is the case we consider here, the first-order and dominating moiré from two identical square gratings is $((\pm1,0,\mp1,0),\ (0,\pm1,0,\mp1))$-index (two perpendicular $(\pm1,\mp1)$-moirés in the 1D case, denoted with subscript 1, 2, 3, 4). $((\pm1,0,\mp1,0)$ has the lowest order and the largest amplitude among all impulses within the visible range (Fig. S20). As $\Psi\in[34^\circ,40^\circ]$, the dominating moiré is $(\pm1,\pm2,\mp2,\mp1)$, the higher order moiré (2nd order).

The period T and the orientation angle ω of the super lattice for a small rotation angle ($\Psi\le 21^\circ$, see Fig. S18A-D) can be understood by geometric moiré theory. The two square lattices of the integer defects can be regarded as four 1D cosinusoidal patterns. When $\Psi\le 21^\circ$, it gives rise to a super lattice shown in Fig. 5B, D. The measured T and ω in simulations and experiments agree well with the geometric moiré pattern theory (Fig. 5E, F, Movie S7), again demonstrating that the disclinations that emerged in the nematic moiré pattern are dictated by the geometry of the pattern. However, the experimental POM images (Fig. 5E, F) of the nematic moiré pattern are different from the isotropic moiré pattern (Fig. 5B), again showing the uniqueness of our proposed nematic moiré.

At large rotation angles, $\Psi\ge 21^\circ$, the periodicity of the disclinations in the nematic moiré pattern is untrackable (Fig. S18E, F). This is consistent with the geometric theory of the moiré pattern, which shows that there is no dominating frequency for $\Psi\in[21^\circ,34^\circ]$ and the frequency of the $(1,2,-2,-1)$-moiré becomes comparable with that of the $(1,0,-1,0)$-moiré for $\Psi\in[34^\circ,40^\circ]$ (Fig. S20) [7], frustrating the nematic and rendering it a less ordered defect structure. As $\Psi\in[34^\circ,40^\circ]$, the dominating moiré is $(\pm1,\pm2,\mp2,\mp1)$, the higher order second moiré. At $\Psi=\arctan(3/4)\approx 36.8699^\circ$, the period T is infinitely large (as plotted in the blue region in Fig. 5G) and therefore it's a singular point [7]. Comparing Fig. S20A and B, we can see that the 1st order moiré is more visible than the 2nd order moiré. In the simulation and experiment, we cannot find periodic defect structures exhibiting the moiré period T (the period is plotted in the blue region in Fig. 5G) within $\Psi\in[34^\circ,40^\circ]$. Therefore, we are not able to compare the moiré theory to the simulation and experiment in the blue region ($\Psi\in[34^\circ,40^\circ]$) in Fig. 5G and Fig.5H.

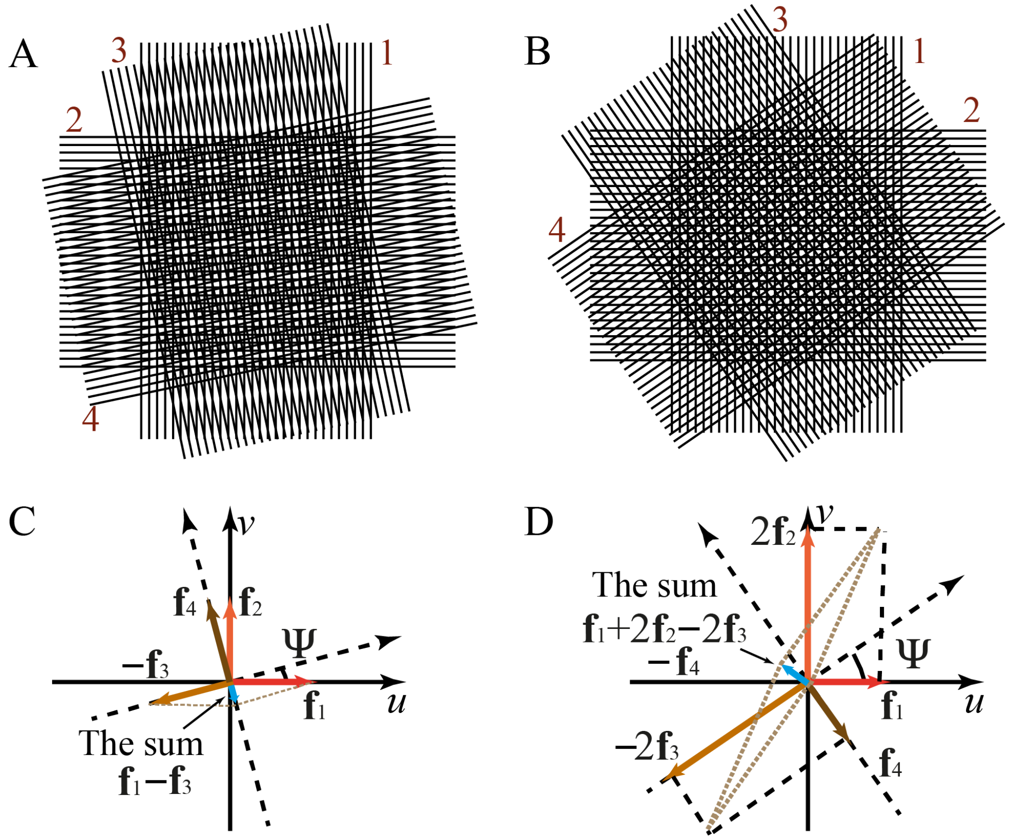

Figure S20: **Geometric moiré in square grids with** $T_1 = T_2 = T_3 = T_4$. (A) $(1, 0, -1, 0)$-moiré dominates at $\Psi = 15°$. (B) $(1, 2, -2, -1)$- moiré dominates at $\Psi = 34°$. Numbers 1–4 represent the four layers of the 1D gratings, where Layer 1 and Layer 2 are for the bottom square pattern and Layer 3 and Layer 4 constitute the other top, rotated square pattern. (C) and (D), vector diagram of spectral interpretation of minimized moiré impluses at $\Psi = 15°$ and $\Psi = 34°$.

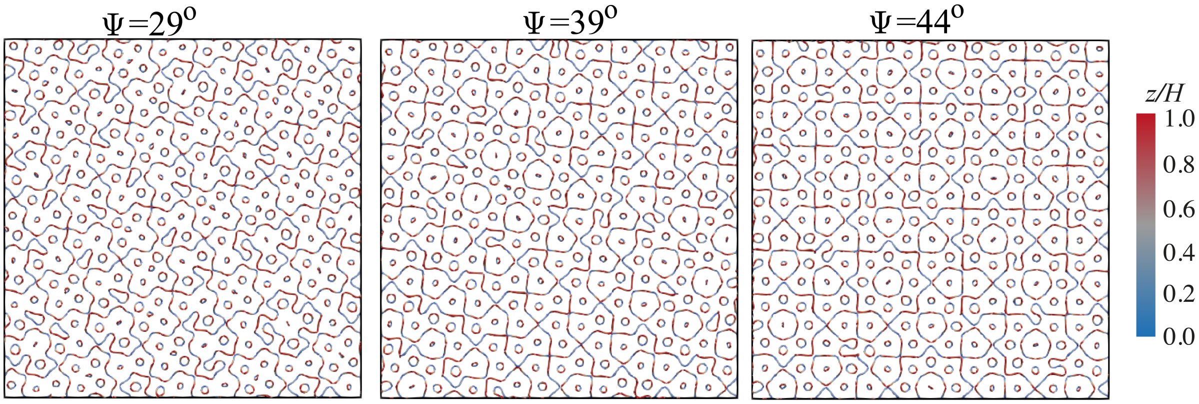

Figure S21: **Aperiodic defect configurations at** $\Psi = 29°$**,** $39°$ **and** $44°$.

T and ω of the 2D square lattice are depicted in Fig. 5G and Fig. 5H. Fig. 5G and H use yellow color for the $\Psi \in [0°, 21°]$ regime and blue color for the $\Psi \in [34°, 40°]$ regime, The two regimes have different dominating modes. For the other angle ranges, $\Psi \in [21°, 34°]$ and $\Psi > 34°$, no dominating moiré is obtained [7] and defects show aperiodic configurations (Fig. S21), the same as the geometric moiré [7].

3.5 Director field at $\Psi = 36.8°$

Fig. S22 shows the defect configuration at $\Psi = 36.8°$, including the top and bottom pattern of a periodic defect configuration. By looking at the two patterns, we see there are two $+1$ defects overlapped at its center, four -1 defects overlapped at its four corners. In its center, the $+1$ defect has rotational symmetry, so the loop connecting two $+1$ defects are identical with the $\Psi = 0°$ defect loop in Fig. S17. The four loops connecting -1 defects are twisted.

3.6 The effect of rotation center

All the 2D defect lattice pattern analysis in this study has used a fixed rotation center, i.e., at a -1 defect. However, we find the rotation center can influence defect period and configuration at $\Psi = \arctan(3/4)$ (Fig. S23). Assume the $x - y$ coordinate of the rotation center is (C_x, C_y). As for a small angle Ψ, from translating top substrate results (Movie S12) and different trials in Fig. S24), we learn that the nematic moiré preserves the $(\pm 1, 0, \mp 1, 0)$-moiré period and the tilting angle. Therefore when $\Psi \leq 21°$, the choice of the rotation center won't affect the defect configuration.

3.7 Statistics of the quasi-loops

In this work, we test the effect of the rotation speed in the 2D ± 1 defect pattern (Fig. 4K). We characterize the simulation results of different rotation speeds (different simulation step N)

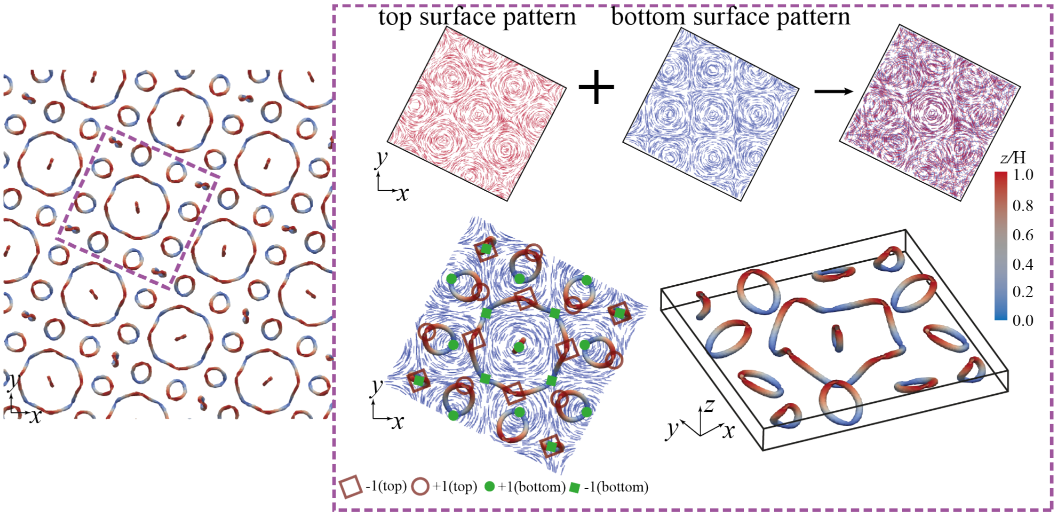

Figure S22: **Defect details for $\Psi = \arctan(3/4)$.** The left is the whole disclination structure with the periodic defect structure labelled. The right is enlarged of the configuration, anchoring field and mid plane ($z/H = 0.5$) director field. ± 1 defect cores on both substrates are noted.

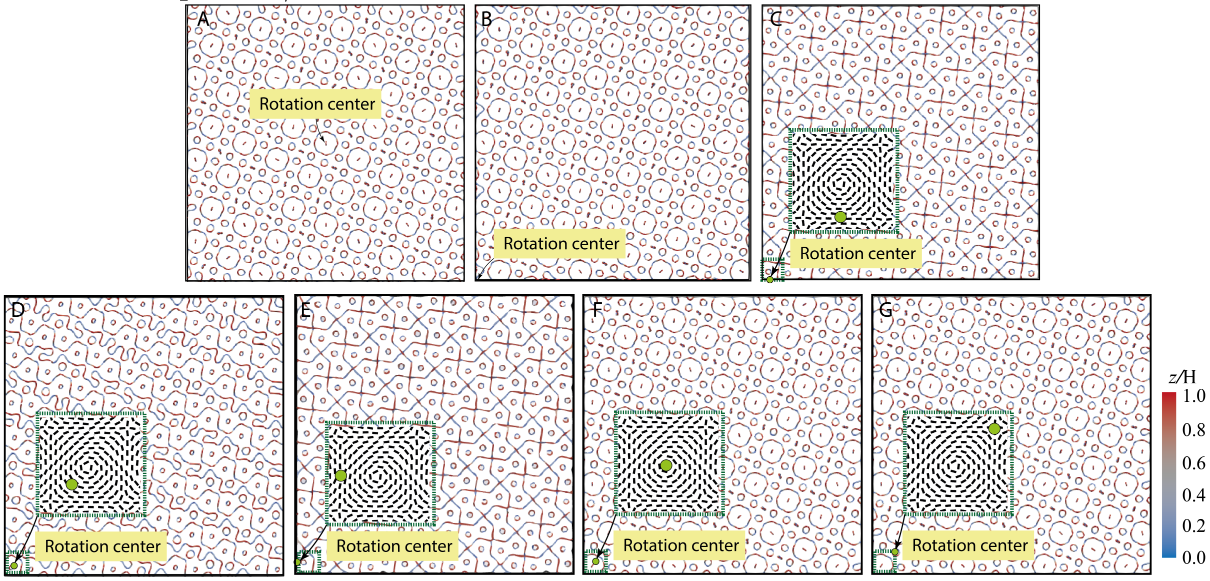

Figure S23: **Defect configurations of three rotation centers at $\Psi = \arctan(3/4)$.** Inset is a schematic showing the rotation center point. (A) The center of the box as the rotation center. (C_x, C_y) is the coordinate of the rotation center. (B) The -1 defect at the corner as the rotation center, $(C_x, C_y) = (0, 0)$. (C) A point in the middle of two neighboring $+1(-1)$ defect pairs, $(C_x, C_y) = (L/2, 0)$. (D) A point in the middle of a $+1$ and a -1 as the rotation center, $(C_x, C_y) = (L/4, L/4)$. (E) Another point in the middle of two neighboring $+1(-1)$ defect pairs, $(C_x, C_y) = (0, L/2)$. (F) A $+1$ defect as the rotation center, $(C_x, C_y) = (L/2, L/2)$. (G) Another -1 defect as the rotation center, $(C_x, C_y) = (L, L)$.

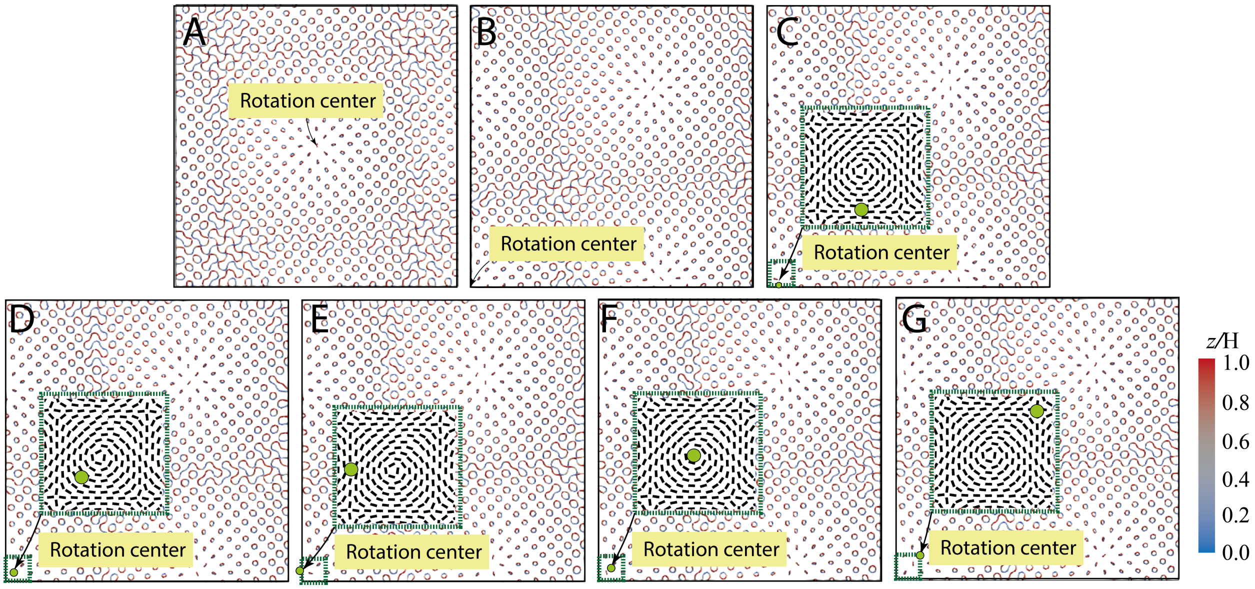

Figure S24: **Defect configurations of three rotation centers at $\Psi = 5°$.** Inset is a schematic showing the rotation center point. (A) The center of the box as the rotation center. (B) The -1 defect at the corner as the rotation center, $(C_x, C_y) = (0, 0)$. (C) A point in the middle of two neighboring $+1(-1)$ defect pairs, $(C_x, C_y) = (L/2, 0)$. (D) A point in the middle of a $+1$ and a -1 as the rotation center, $(C_x, C_y) = (L/4, L/4)$. (E) Another point in the middle of two neighboring $+1(-1)$ defect pairs, $(C_x, C_y) = (0, L/2)$. (F) A $+1$ defect as the rotation center, $(C_x, C_y) = (L/2, L/2)$. (G) Another -1 defect as the rotation center, $(C_x, C_y) = (L, L)$.

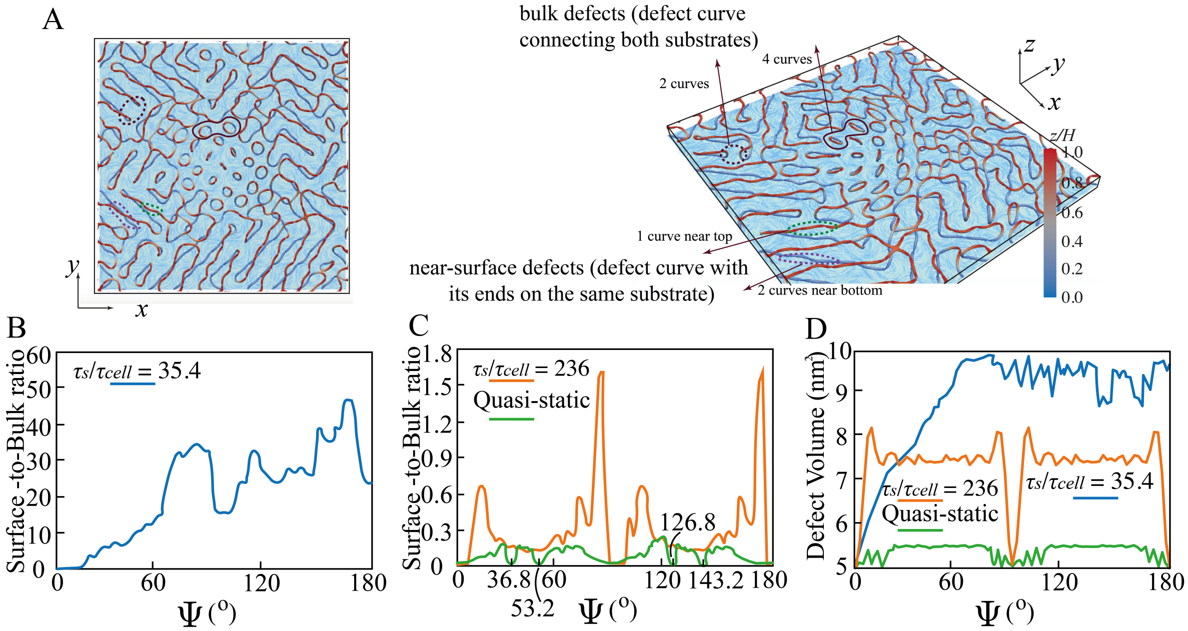

Figure S25: **The ratio of defect curves near the surfaces and defect curves through the cell.** (A) A planar view and side view of a fast rotation with two kinds of defects labeled. The ratio of the number of defects near the surfaces to the number of defects going through the cell changes with the rotation angle Ψ: (B) $\tau_s/\tau_{\text{cell}} = 35.4$ (rate-I); (C) $\tau_s/\tau_{\text{cell}} = 236$ (rate-II) and quasi-static. (D) Total volume change of defects of the three cases. Source data are provided as a Source Data file.

here in Fig. S25. Those quasi-loops in fact consist of two separate curves, as presented clearer in Fig. 5E and F. During substrate rotation, defect creation/annihilation may occur. However, since each defect core on the two patterns can generate two split curves, the total number of defect curves is constant during the rotation. Here we count the ratio between bulk defects (defect curve connecting both substrates) and near-surface defects (defect curve with its two ends on the same substrate). From Fig. S25B and C, we learn that when the rotation is sufficiently fast, the system doesn't have time to relax to satisfy new anchoring pattern, and there are more near-surface defects. Since we apply $H/L = 0.3$ in the simulation, defects are shorter if they go through the bulk from top to bottom. In quasi-static simulations with the system remaining in the stable state, more defect lines choose to connect through the bulk (Fig. S25B, C), and as a result, its defect volume is lower than $\tau_s/\tau_{\text{cell}} = 35.4$ and $\tau_s/\tau_{\text{cell}} = 236$ (Fig. S25D). Here, τ_{cell} is the characteristic time scale for the relaxation of the nematic director field, $\tau_{\text{cell}} = (\gamma_1 H^2)/L_1 = (2S^2H^2)/(\Gamma_s L_1)$ with γ_1 being the rotational viscosity, H being the cell gap, and L_1 being the elastic constant. The simulation time $\tau_s = N\tau_0$ is expressed in the simulation time unit τ_0 (N is the number of simulation steps). To achieve a quasi-static simulation, $\tau_s \gg \tau_{\text{cell}}$ has to be satisfied.

4 Reconfiguration of nematic moiré pattern under an electric field

To explore the potential application in LC display, we study the response of the nematic moiré pattern to a static electric field. A uniform nematic cell can undergo the so-called Frederiks transition if an electric field applied normal to the cell surface is above a threshold value [10]. For a nematic with positive dielectric constant anisotropy, namely $\epsilon_{\parallel} > \epsilon_{\perp}$, where $\epsilon_{\parallel}$ and $\epsilon_{\perp}$ respectively represent the dielectric constant parallel and perpendicular to $\mathbf{n}$, the director field tends to rotate parallel to the electric field $\mathbf{E}$ during the Fredericks transition, resulting in a change of its optical appearance. This electro-optical response serves as the basic mechanism for display technology.

4.1 Frederiks transition in the twist cell

In a planar twisted nematic cell (TNC), there are two uniform planar anchorings on each of the two substrates ($z = 0$ and $z = H$), and their angle difference is Ψ_0. In the unperturbed state, θ only varies with z, $\theta(x, y, z) = \Psi_0 z/H$. The deduced transition threshold is [10]

$$E_c^2(\Psi_0) = E_c^2(0)\left[1 + \frac{K_3 - 2K_2}{K_1}\left(\frac{\Psi_0}{\pi}\right)^2\right], \tag{S10}$$

where $E_c(0)$ is the Frederiks transition threshold for a uniform nematic cell. Under one-constant assumption ($K_1 = K_2 = K_3 = K$) in the simulations. Therefore, Eq. S10 reduces to

$$E_c^2(\Psi_0) = E_c^2(0)\left[1 - \left(\frac{\Psi_0}{\pi}\right)^2\right],$$

where $E_c(0) = \frac{\pi}{H}\sqrt{K/\epsilon_0\Delta\epsilon}$, with $\Delta\epsilon$ the dielectric constant difference. In this work, the cell thickness is H, and the transition voltage for the planar twisted cell and uniform cell are $V_c(\Psi_0) = HE_c(\Psi_0)$ and $V_c(0) = HE_c(0)$, respectively.

4.2 The Frederiks transition in the nematic moiré

To characterize the Frederiks transition in nematic moirés, we introduce a tilting angle $\phi = \cos^{-1}\mathbf{n}_z$ to represent the angle between $\mathbf{n}$ and the xy-plane. Because the surface preferred director orientations are within the xy-plane, $\phi = 0$ when $\mathbf{E}$ is below a threshold value E_c. When $\mathbf{E} = E\mathbf{z}$ with $E > E_c$ is applied, the director starts to rotate towards $\phi = \pi/2$. Here, we consider the 1D cusp-like pattern and compare the average tilting angle $\bar{\phi}$ in the midplane of the cell as a function of the applied voltage V for the three defect states with the same cell gap H in the simulation (Fig. S26A). We use 30 lattice points in the simulation and therefore $H \approx 30 \times 6.63$ nm $= 198.9$ nm. The relaxed structure in the absence of an electric field

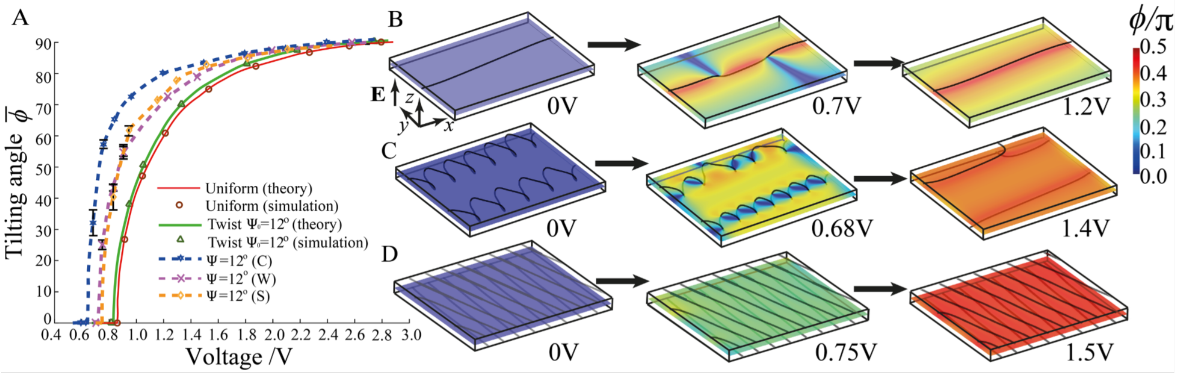

Figure S26: **Frederiks transition of uniform cell, twist cell, and 1D cusp-like splay-bend nematic moiré cells**. (A) The averaged director tilting angle $\bar{\phi}$ in the midplane of the cell versus electric voltage change with fixed cell gap H for different defect states formed at a fixed rotation angle $\Psi = 12^o$. Solid lines correspond to the results from planar cells and planar twisted nematic cells at the same twisting angle 12°. Source data are provided as a Source Data file. The reconfiguration process and the middle plane tilting angle ϕ/π of the S-state (B), C-state (C) and W-state (D). We use 30 lattice points as the fixed cell gap for three states. The lattice constants (L) for the S-, C-, and W-state are 150×6.63 nm ≈ 994.45 nm, 60×6.63 nm ≈ 397.8 nm, and 30×6.63 nm ≈ 198.9 nm, respectively.

(Fig. 1) all have $n_z = 0$ component since (1) we apply the initial condition with $n_z = 0$, and (2) our infinite anchoring is planar anchoring. We added an initial noise to the director field before applying the electric field. Specifically, we added a small random number Δn_z uniformly distributed in $[-10^{-10}, 10^{-10}]$ to the z component of all the bulk LC points and normalize the director **n** to allow the Frederiks transition to happen.

Twisting can lower the threshold voltage (Eq. S10). For comparison purpose, we choose $\phi_0 = \phi = 12^o$ for both twisted nematic cells (TNCs) and nematic moiré cells. The measured $\bar{\phi}$ for both uniform cells and TNCs in the simulations agree well with the above theory (Fig. S26A). Similar to the effect of twisting in planar cells, spatial distortions of the nematic imposed by the surface patterning can also facilitate the onset of the Frederiks transition (Fig. S26B). Indeed, the nematic moiré pattern exhibits a threshold voltage approximately 10% lower than that of TNCs, showing the promise of nematic moiré patterns in applications such as displays and responsive materials.

With the cell gap H fixed, for different defect states corresponding to different pattern periodicities L, V_c is different among them. Interestingly, the C-state showing the helical-like disclinations corresponds to an intermediate L but exhibits the lowest V_c (Fig. S26A). This non-monotonic behavior in terms of the pattern periodicity can be understood in the following: in general, regions with more twist distortions have a smaller transition threshold; therefore, it is expected that the C-state (with a smaller L) has a lower V_c than the S-state. For the W-state,

however, disclinations are formed near the surfaces, and its nematic director in the bulk is almost uniform (Fig. S4), which significantly reduces its twist frustrations, therefore giving rise to a V_c higher than that of the C-state.

The director rotation in response to the electric field is spatially heterogeneous in the nematic moiré cells. We expect that the transition is initiated from regions of the highest twist distortions. For the S-state, this corresponds to the regions near the disclinations, where ϕ is closer to $\pi/2$ than that in defect-free regions for intermediate and high voltages (Fig. S26B). For the C-state, however, the highest twist distortions are located in the defect-free regions. Twist distortions are released by generating defects in regions occupied by near-surface disclinations (Fig. S3). This is reflected by the fact that ϕ approaches $\pi/2$ earlier in regions not occupied by the disclinations (Fig. S26C). As the voltage increases, the radius of the helical-like disclinations shrinks. In the meantime, they are repelled from the bulk and reconfigured near either the top or the bottom substrate in the form of straight lines (Fig. S26C). The W-state has small twist distortions in the bulk and does not show spatial heterogeneity in terms of the director rotations (Fig. S26D). At the highest voltage, straight disclinations in all the states vanish, and the directors are all aligned by the electric field.

4.3 The responses at different angles

At different rotation angle Ψ, the S-, C- and W-state of the 1D cusp-like splay-bend pattern exhibit different responses under the electric field. We can understand the behaviors since the transition is initiated from regions of the highest twist distortions. The W-state has small twist distortions in the bulk and therefore doesn't show spatial heterogeneity in terms of director rotations, so the transition behavior doesn't vary with pattern rotation angle Ψ (Fig. S27). The C-state initiates transition in the defect-free regions, and as with the increase of Ψ, moiré period T decreases, and the number of defect curves in the cell increase, so the defect-free volumes reduce and transition behavior becomes harder Fig. S28. The S-state has larger twist in the defect regions comparing to its defect-free regions, so as with the increase of Ψ , the number of defects increases and the transition behavior becomes easier Fig. S29.

5 Additional details of the methods

5.1 Experiment details

The materials used in the experiments are given in Fig. S30. Anchoring patterns are made by photopatterning technique, using 2D ±1 defect pattern as an example (Fig. S31A-D). One period L of the designed 1D cusp-like splay-bend pattern is in Fig. S32.

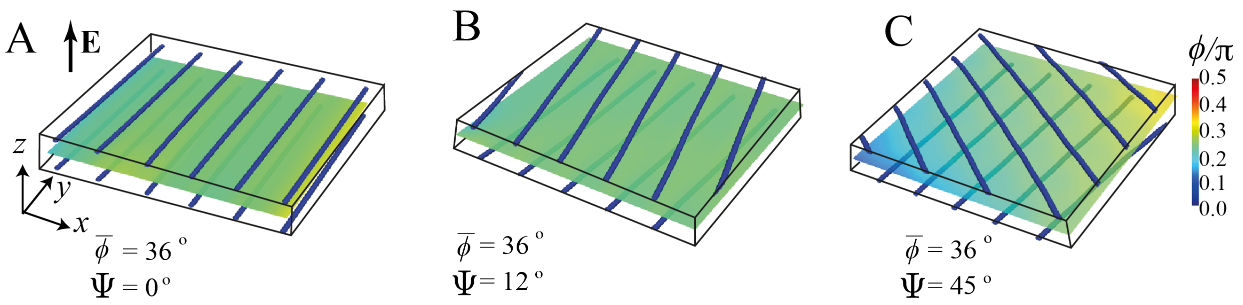

Figure S27: **Electric Field ($|\mathrm{V}| = 0.82$ V) induced transition of the W-state ($H/L = 1$) at different rotation angles.**

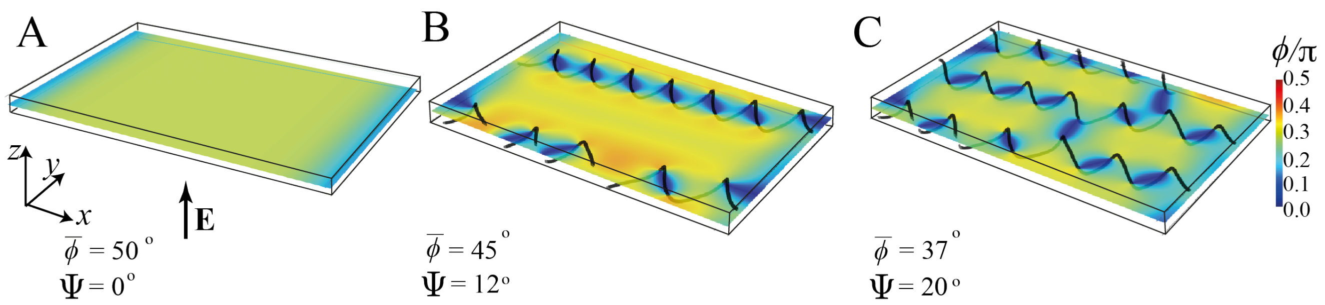

Figure S28: **Electric Field ($|\mathrm{V}| = 0.69$ V) induced transition of the C-state ($H/L = 0.75$) at different rotations.**

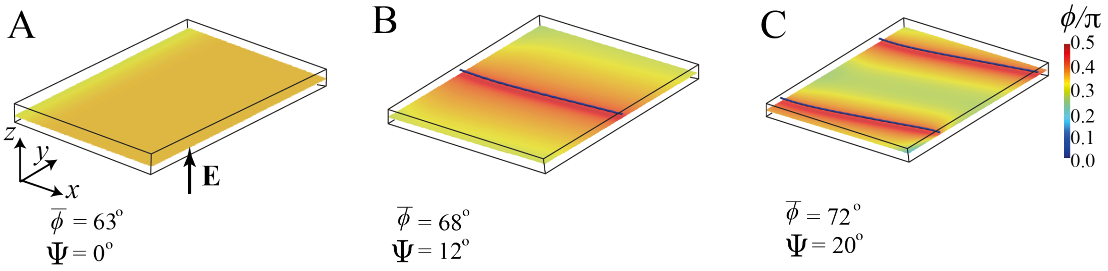

Figure S29: **Electric Field ($|\mathrm{V}| = 0.95$ V) induced transition of the S-state ($H/L = 0.25$) at different rotation angles.**

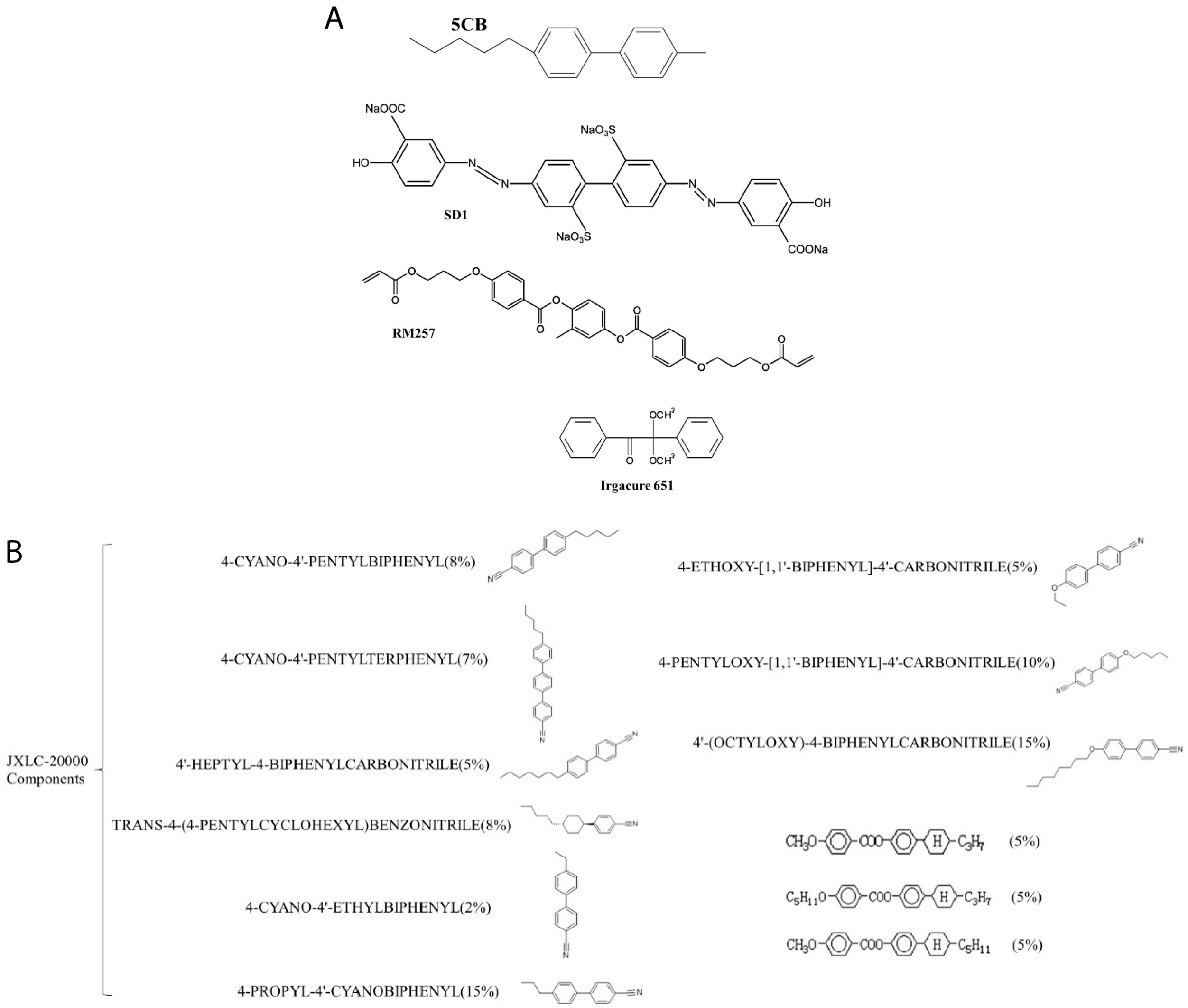

Figure S30: **Chemical structures of used materials in the experiments.** (A) From top to bottom: chemical structure of 5CB, SD1, RM257, and Irgacure 651. (B) Chemical structures of the components of the LC mixture JXLC-20000.

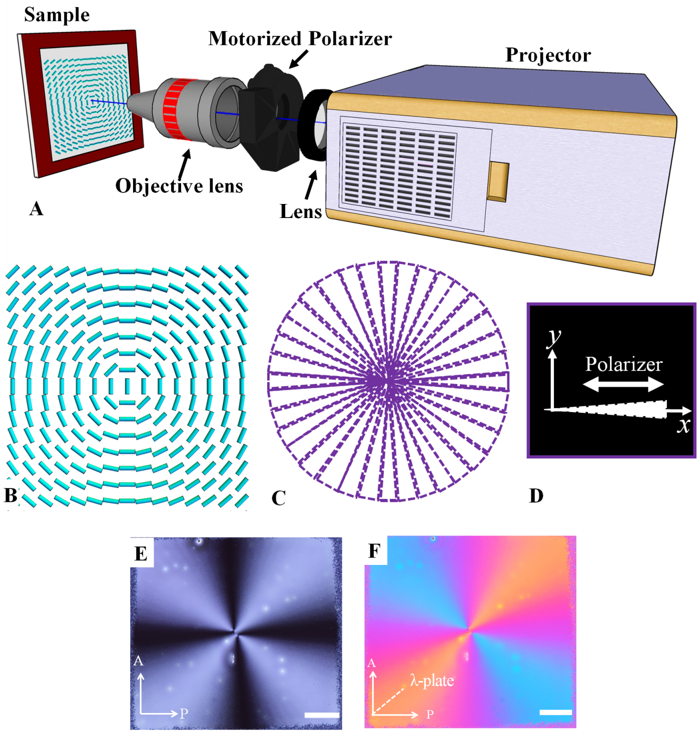

Figure S31: **Maskless photopatterning setup**. (A) Schematic of maskless photopatterning setup. (B) Director field of the circular $+1$ defect; (C) The circular pattern is divided into 36 segments; (D) Polarizer follows x-axis at the beginning; (E)-(F) Polarizing micrographs of the designed pattern. Scale bar: $50\ \mu$m.

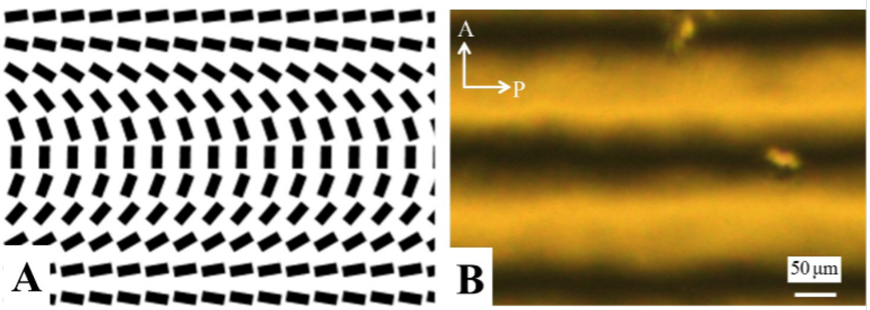

Figure S32: **1D cusp-like splay-bend pattern**. Designed director field of 1D cusp-like splay-bend pattern (A) and the corresponding polarizing micrograph (B). Scale bar: $50\ \mu$m.

5.2 Simulation details

5.2.1 Modeling details

More straightforwardly, elastic energy is written in the Frank–Oseen form [11]

$$\begin{aligned} f_{el}^{FO} =& \frac{1}{2}K_1(\nabla\cdot\mathbf{n})^2 + \frac{1}{2}K_2(\mathbf{n}\cdot\nabla\times\mathbf{n})^2 + \frac{1}{2}K_3(\mathbf{n}\times(\nabla\times\mathbf{n}))^2 \\ &- \frac{1}{2}K_{24}\nabla\cdot[\mathbf{n}(\nabla\cdot\mathbf{n}) + \mathbf{n}\times(\nabla\times\mathbf{n})], \end{aligned} \tag{S11}$$

where K_1, K_2, K_3 and K_{24} are splay, twist, bend, and saddle-splay moduli, respectively. For consistency, alternatively, elastic energy density f_{el} in the simulation is expressed in terms of $\mathbf{Q}$-tensor form as

$$\begin{aligned} f_{el}^{Q} =& \frac{1}{2}L_1(\partial_k Q_{ij})(\partial_k Q_{ij}) + \frac{1}{2}L_2(\partial_k Q_{jk})(\partial_l Q_{jl}) + \frac{1}{2}L_3 Q_{ij}(\partial_i Q_{kl})(\partial_j Q_{kl}) \\ &+ \frac{1}{4}L_4(\partial_l Q_{jk})(\partial_k Q_{jl}). \end{aligned} \tag{S12}$$

The mapping between constant sets K_1, K_2, K_3, K_{24} and L_1, L_2, L_3, L_4 is

$$\begin{aligned} L_1 &= \frac{1}{2S_0^2}[K_2 + \frac{1}{d}(K_3 - K_1)], \\ L_2 &= \frac{1}{S_0^2}(K_1 - K_{24}), \\ L_3 &= \frac{1}{2S_0^3}(K_3 - K_1), \\ L_4 &= \frac{1}{S_0^2}(K_{24} - K_2), \end{aligned} \tag{S13}$$

where d is the dimensionality of the system. We use one-constant assumption ($K_1 = K_2 = K_3 = K_{24}$). The disadvantage of the simulation is that the size is much smaller compared to the experiment. The characteristic length scale of the simulations is set to 6.63 nm (nematic coherence length). We use 30 lattice points for the period L and thus $L = 30 \times 6.63$ nm $= 198.9$ nm. The simulation box in xy plane has the size of 420 (length) $\times 600$ (width), with the characteristic length $\xi_N = \sqrt{L/A_0} \approx 6.63$ nm. Box thickness is varied. Considering the figure size, the results are usually cut from the simulation box. Instead of matching the absolute length scale, we compare simulations and experiments at the same H/L ratio. Despite the mismatch in system size, simulation and experiment agree very well in terms of defect structures and optical patterns. We do not apply noise during the simulation but use different ansatzes to account for the effect of thermal noise.

We do not impose periodic boundary conditions since the rotated pattern does not have translational symmetry. We instead use free boundary conditions (zero anchoring) for the sidewalls, which will inevitably induce inhomogeneities near the sidewalls. This boundary effect

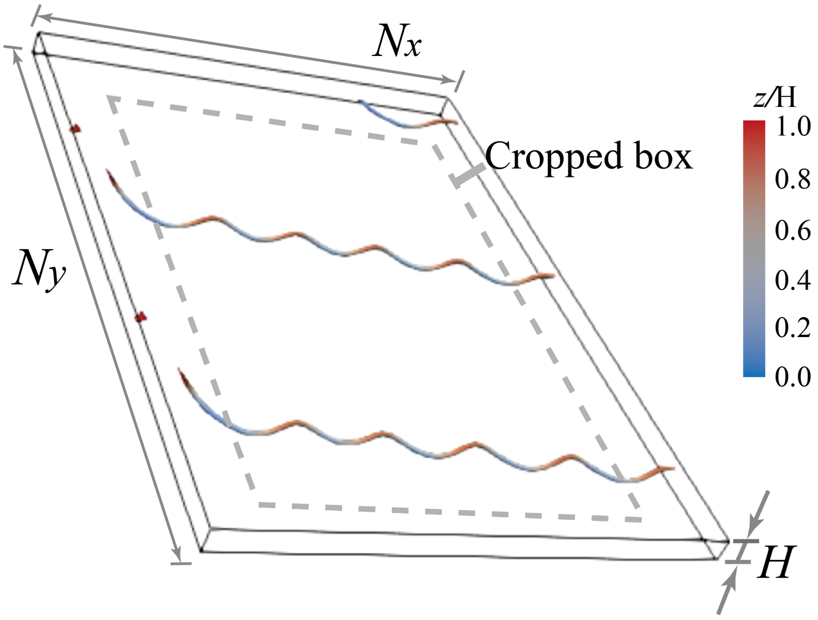

Figure S33: **The full simulation box for the 1D cusp-like and sinusoidal pattern with the cropped box shown as a dashed box**. Except for the electric field calculations (Sec. 5 in the Supplementary Information), $N_x = 420$ and $N_y = 600$ in simulation, and H is varied between 3 to 40. Pattern constant L is set to 30. The cropped box has 300 lattice points in the x dimension and 300 lattice points in the y dimension.

does not impact the nematic structure near the center of the simulation box. When showing the defect configurations, we manually cut the distorted regions near the sidewalls. In Fig. S33 we show the actual simulation box and the cropped box.

For the 1D cusp-like splay-bend pattern and 1D sinusoidal splay-bend pattern, the box width direction has 420 lattice points (N_x), and the length direction has 600 lattice points (N_y). The thickness is varied between 3 and 40 lattice points. We fix lattice constant L as 30 points except for the electric field results (the parameters are in the caption of Fig. S26). The size of cropped box is 300 by 300 in $x - y$ dimension for the 1D cusp-like and sinusoidal pattern. The simulation results without the cropped box can be found in Movie S1. For the 2D ± 1 defect pattern, there are 18 periodic units (Fig. 5A) along both x and y direction in the simulation box, with $H/L = 0.3$ and $L = 30$. The simulation results without cropping are shown in Movie S11. In Fig. 5D and 5I, the cropped box size is 13 by 13, 7 by 7, respectively.

We set infinite (or fixed) anchoring condition for both top and bottom substrate in the simulation to approximate the strong anchoring condition in the experiment. The patterned surface in the experiment has a well-defined easy-axis pattern, which becomes discontinuous at defect points (Fig. S34). Therefore, we set all the surface points to have the equilibrium bulk value $S_0 \cong 0.62$. The **Q**-tensor of the surface points is not evolved in the simulation. Bulk points next to the discontinuity points on the surface will have depressed order parameter due to the diverging director field. The disclinations that emerged in the simulation are essentially bulk line defects, with their ends connected to those discontinuity points. We further show that we

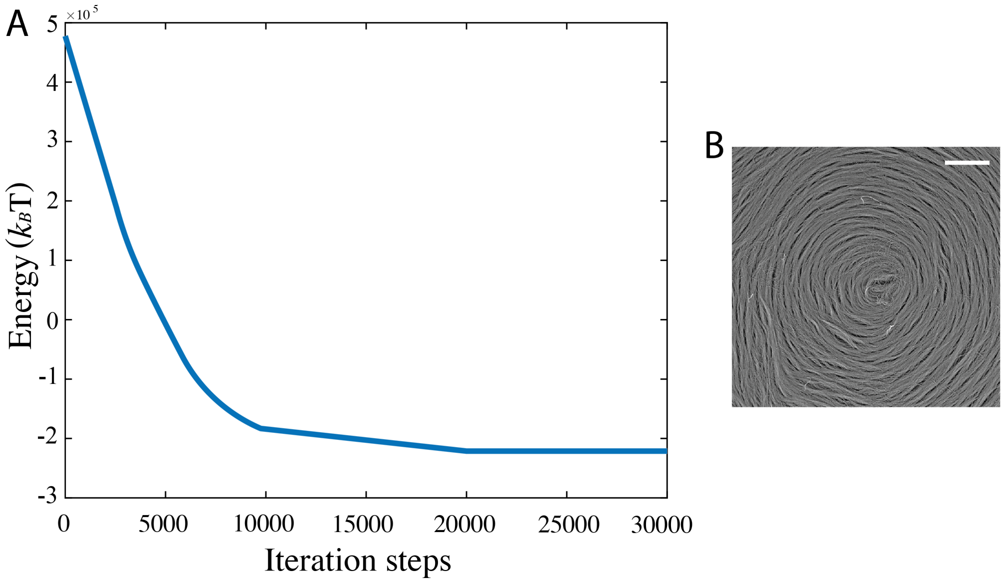

Figure S34: **More details of ±1 defect pattern.** (A) Free energy convergence process in the ±1 defect pattern simulation (at $\Psi = 20°$). Source data are provided as a Source Data file. (B) Scanning electron microscope (SEM) experimental image of the $+1$ defect pattern adopted from [16]. Scale bar: $30\ \mu$m.

do not encounter divergence issue in the simulation. For example, the ±1 defect pattern at $\Psi = 20°$ exhibits free energy convergence (Fig. S34). The good match between the experiment and simulation (Fig. 5I, J) further validates our simulation method.

Because the system size in simulation is much smaller compared to the experiment, while the defect core size cannot be scaled, the defect lines appear thicker in the simulated optical images than in the experimental optical images, regardless of what optical simulation method we use, as seen in Fig. 3H, 3I, Fig. S6, and S11. Because in the simulation, the ratio between the defect core size and pattern periodicity is $\xi_N/L = 1/30$, which is much smaller than 1, the presence of disclinations does not impact the bulk nematic structure, as evidenced by the good match between experimental and simulated optical images. There are indeed some subtle differences between the two images, which are attributed to the difference in size. Therefore, the system size effect may play a role in the optical applications of nematic moirés. This is beyond the scope of the current study and we leave it for the future work.

5.2.2 Initial director fields in the simulations

To find the true ground state with global minimum, instead of using quasi thermal noise, we compare different initial conditions in the simulation. Among the three defect types, the S-state has the strongest patterning effect, and therefore the shape in equilibrium is insensitive to initial conditions; on the other hand, the W-state is sensitive to its initial condition in the simulation. We have compared four different conditions in Table S1(at $\Psi = 12°$, $H/L = 0.9$), and the corresponding defect structure and the mid plane director in Fig. S35. The first three initial conditions have uniform bulk director, $\theta = 0$, $\theta = \pi/2$, and $\theta =$ (θ is the in-plane tilting angle of the director with the x-axis). From the comparison, initial condition $\theta = 0$ can give rise to

the lowest free energy, so we adopt it as the initial condition in our simulations. The simulation box for Table S1 is 420 (length, N_x) $\times$600 (width, N_y) $\times$29 (height, H) (see Fig. S33) with the characteristic length $\xi_N = \sqrt{L/A_0} \approx 6.63$ nm.

Table S1: **Total energies ($k_{\mathrm{B}}T$) of the W-state from different initial conditions**

Initial	$\theta = 0$	$\theta = \pi/2$	$\theta = \pi/4$	random
Total energy (simulation units)	-14968.8	-14959.2	-14930.5	-14928.6

As for the C-state, the four cases all give rise to helical-like defect curves (Fig. S36). Their energies are close (comparing with the W-type) in Table S2, and the random initial condition shows the lowest energy. The two rhombuses give director near the two defect curves. The simulation box for Table S2 is $420 \times 600 \times 15$ ($\xi_N = \sqrt{L/A_0} \approx 6.63$ nm).

Table S2: **Total energies ($k_{\mathrm{B}}T$) of the C-state from different initial conditions**

Initial	$\theta = 0$	$\theta = \pi/2$	$\theta = \pi/4$	random
Total energy (simulation units)	-7701.7	-7699.9	-7701.7	-7707.5

Like the 1D cusp-like pattern, different initial conditions in sinusoidal pattern are also tested to obtain the ground the state for the thick cell. $\theta = \pi/2$ condition has the minimal energy. We did not add quasi-thermal noise in the simulations. Instead, to get the actual relaxed state with minimized energy and avoid being stuck in the local minimum, we try different initial conditions (ansatz) as a way to test the system's reaction to noise.

5.2.3 The rotation speed for fast rotation simulations

In the work, we perform quasi-static rotation if there's not a special notice. When the system completes a π-turn ($\Psi = \pi$) in the time τ_{s} that is much longer than the characteristic relaxation time of the nematic cell $\tau_{\text{cell}} = \frac{\gamma_1 H^2}{L_1}$ with γ_1 the rotational viscosity of the nematic and L_1 the elastic modulus, the process is quasi-static, and the system can recover its initial state in which disclinations simply connect overlapping defects. However, when the rotation rate is fast enough (the simulation time τ_{s} is short), the rotation operation can fundamentally change the emerging defect pattern. In simulations, we first relatively rapidly rotate the top substrate with ($\tau_{\mathrm{s}}/\tau_{\text{cell}} \approx 35.4$), and disclinations appearing as parallel line segments connecting surface defects from the same surface are more favored (Fig. 5K, rate 1 in Movie S13). During fast rotations, the bulk nematic does not have enough time to relax back to its true equilibrium state and is therefore stuck into a more uniform director field, leading to the formation of near-surface disclinations (rate 1 in Movie S13). As the rotation speed becomes slower ($\tau_{\mathrm{s}}/\tau_{\text{cell}} \cong 236$),

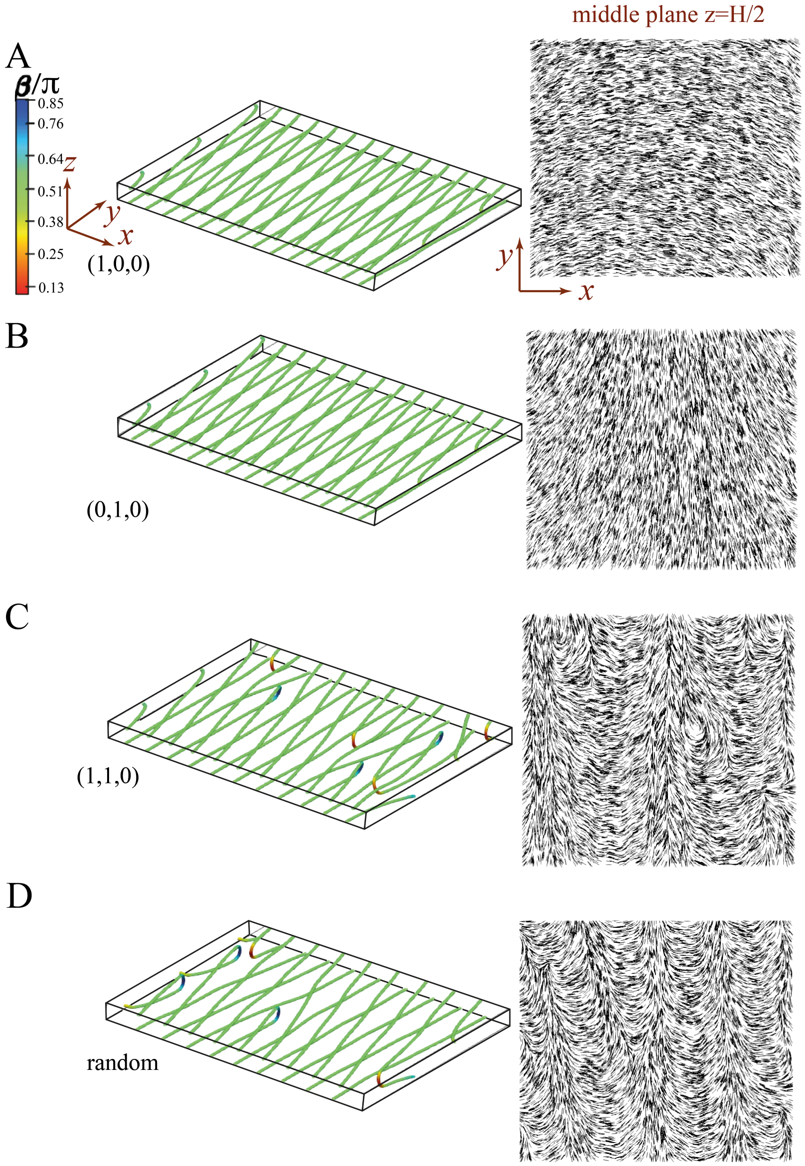

Figure S35: **Four different initial conditions and the four resulting defect structures of the W-state** ($H/L = 0.9$).

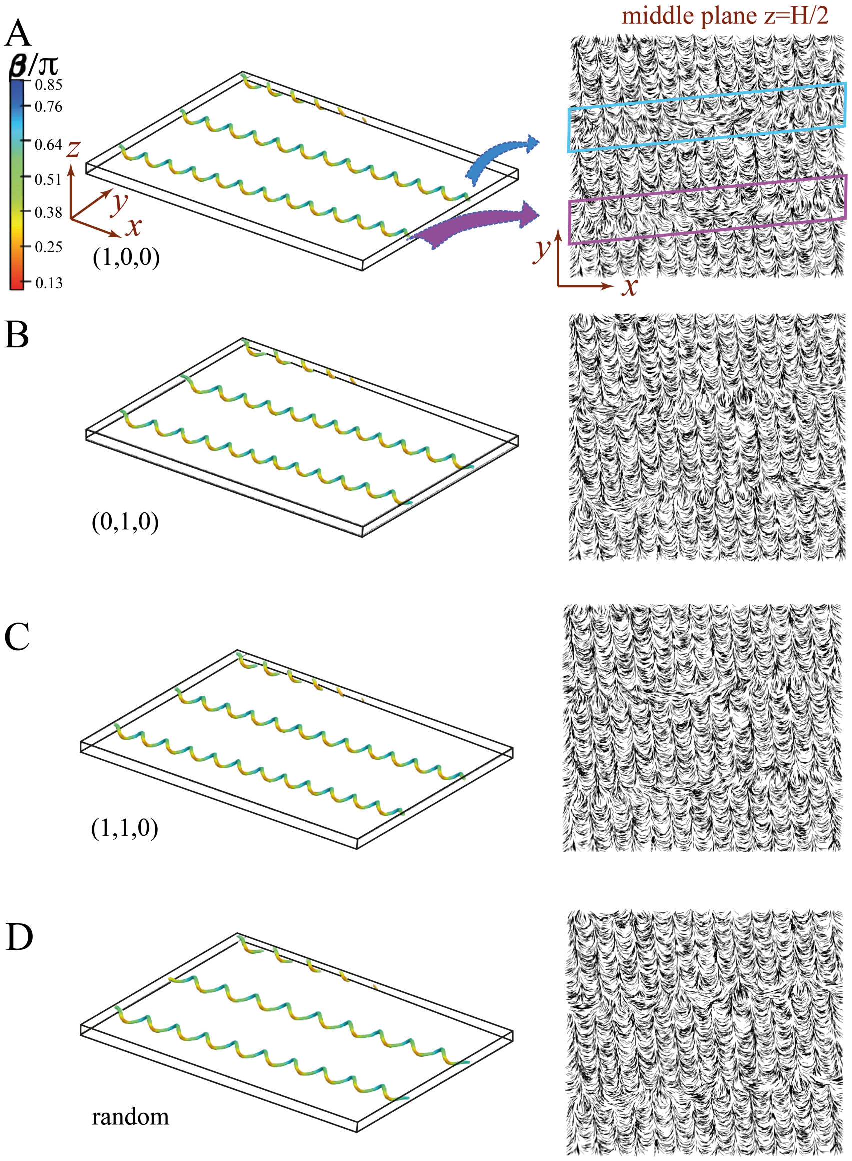

Figure S36: **Four different initial conditions and the four resulting defect structures of the C-state** ($H/L = 0.5$). The two frames corresponds to defect regions (in the xy plane).

the emerging defect configurations will be more similar to those formed during quasi-static rotations (Movie S11). In the fast rotation experiments, periodic defect structures are also absent due to a strong back-flow effect; the disclinations are more irregular and stretched by the flow. Such a hydrodynamic effect will be studied in our future work.

5.2.4 Effects of LC elastic constants

For simplicity, we apply the one-constant assumption (Sec. 5.2.1, $K_1 = K_2 = K_3$) in this work. However, the deformations near state boundaries strongly depend on the anisotropy of the elastic constants, K_1, K_2, and K_3. To show how the state boundaries depend on the elastic constants, we perform simulations using 5CB elastic constants, $K_1 = 6$ pN, $K_2 = 3.9$ pN, $K_3 = 8.2$ pN, and $K_{24} = 7$ pN at $\Psi = 12°$ for the 1D cusp-like splay-bend pattern. We fix the rotation angle Ψ at $\Psi = 12°$ and vary the cell gap H.

When the cell gap is narrow and the surface patterning dominates, both of the two sets of elastic constants show the transition between S-state and C-state at $H/L = 0.35$. Since 5CB has a lower twist elastic constant, twist deformation is the most energetically-favored. From the topological analysis of the S, C, and W-state in Fig. S4, the S-state and W-state exhibit periodic pure-twist local profile and only the twist deformation while the C-state have wedge-twist profile with the bend and splay deformation in the wedge regime. As shown in Fig. S37, the transition between the C-state and the W-state using 5CB constants and one-constant occur at $H/L = 0.62$ and 0.8, respectively. In the C-state, A_{xy}/T varies linearly from 0 to 1 using the one-constant assumption while A_{xy}/T increase slowly for $H/L \in [0.35, 0.6]$ and shows a sudden jump to $H/L = 1$ for $H/L \in [0.6, 0.62]$. The differences of A_{xy}/T variation confirms that the C-state is less energetically favored, if we apply the 5CB elastic constants.

Therefore, the anisotropy of elastic constants can tune the state boundaries and this can be explained by the competition between splay, bend, and twist energies.

5.2.5 Simulation method for generating POM images

In our system, **z** is the incident direction of the wave vector of the polarized light. The light can be written in terms of $\mathbf{V} = \mathbf{x}V_x e^{i\phi_x} + \mathbf{y}V_y e^{i\phi_y}$ [12, 14]. To propagate the polarized state through an optical element (e.g., linear polarizer), we use $\mathbf{V}^{\text{trans}}$ as the transformed state and $\mathbf{V}^{\text{init}}$ as the initial state

$$\mathbf{V}^{\text{trans}} = \mathbf{R}(-\alpha)\mathbf{\Theta}'\mathbf{R}(\alpha)\mathbf{V}^{\text{init}},$$

where the transformation operator $\mathbf{\Theta}'$ is in the form of a 2×2 Jones matrix (in the natural basis) [13, 14]. The natural basis is not always the xy basis, assuming it has an angle α from the $\mathbf{x}$ axis, and $\mathbf{R}_o(\alpha)$ is the rotation matrix

$$\mathbf{R}_o(\alpha) = \begin{bmatrix} \cos\alpha & \sin\alpha \\ -\sin\alpha & \cos\alpha \end{bmatrix}.$$

Discrete the continuous nematic volume into voxel so that in each of the voxel ν of the size Δ, the local director can be treated as constant, $\mu \in [1, H/\Delta]$. LC is birefringent (and uniaxial in this work), with the ordinary index of refraction n_0, and the extraordinary index of refraction is n_e. γ is the angle between $\mathbf{n}$ and the incident direction. n_e can be rewritten as

$$n_e(\gamma) = \frac{n_0 n'_E}{\sqrt{n_0^2 \sin^2(\gamma) + n_E^2 \cos^2 \gamma}},$$

where n'_E is the modified extraordinary index of refraction for $\gamma = \pi/2$ at a LC lattice point (x, y, z), $n'_E(x, y, z) = n_o + (n_E - n_o)S(x, y, z)/0.62$. We apply this modification to improve the simulated image quality by the Jones-matrix approach. Rewrite the Jones matrix $\mathbf{\Theta'}$ in terms of the LC media as

$$\mathbf{\Theta}' = \begin{bmatrix} e^{in_e(\gamma)\frac{2\pi\Delta}{\lambda}} & 0 \\ 0 & e^{in_0(\gamma)\frac{2\pi\Delta}{\lambda}} \end{bmatrix},$$

where λ is the wavelength of the light in vacuum. $\mathbf{z}$ is the incident direction, and the plane of polarization is the $x - y$ plane [13]. Thus, in the final cross-polarized image, the intensity at a given point $I(\boldsymbol{\rho})$ (with $\boldsymbol{\rho}$ being the position vector) [12] is

$$I(\boldsymbol{\rho}) = |\mathbf{\Theta'_A}\mathbf{R}(\alpha_A)\mathbf{v}(\boldsymbol{\rho})\mathbf{E}_p|^2,$$

where $\mathbf{E}_p$ is the orientation of the polarizer, $\mathbf{\Theta}'_A\mathbf{R}(\alpha_A)$ is the operator and associated rotation for the analyzer angle α_A from the $\mathbf{x}$ axis, and

$$\mathbf{v}(\boldsymbol{\rho}) = \prod_{\nu=H/\Delta}^{1} \mathbf{R}_o(-\alpha_\nu)\mathbf{\Theta}'_\nu\mathbf{R}_o(\alpha_\nu).$$

In our approach, we can only get thick dark cores for narrower cells (Fig. S6A-B) with an abrupt change of director field. The simulated defect lines are less visible in thicker cells (Fig. S6C) , where the director changes more smoothly. Note also, Jones matrix-based calculations cannot be used for simulating bright field images. For the Jones-matrix approach, dark areas correspond to the regions where polarized ray in the layer mostly passes areas where the director is either parallel or orthogonal to the polarization. Therefore, a disclination can be indirectly seen if the director field above the line satisfies the mentioned condition. As lines look very thin the director should have a narrow range where this condition is realized. In a Mauguin limit [15] for a very particular director distortion, the polarization can also keep the right orientation. The numerically obtained director field cannot disclose this unfortunately. For thin samples, the Jones-matrix approach is good (Fig. S6A, B) while for the thicker ones where focusing, ray deflection, and oblique rays become relevant, the defect cores are brighter and the director field is less clear to see (Fig. S6C).

In our simulation, the system size is smaller than in the experiment, and the wavelength λ is set to $3.8 \times 6.63 \approx 25.194$ nm to obtain optical images based on the simulation data using the

Jones-matrix approach. We have also performed calculations using the open-source code [8]. For those results, we rescaled our data to the real experimental size and used the experiment wavelength, $400 - 800$ nm. The open-source code can give better results in the thick cell limit, as shown in Fig. S38.

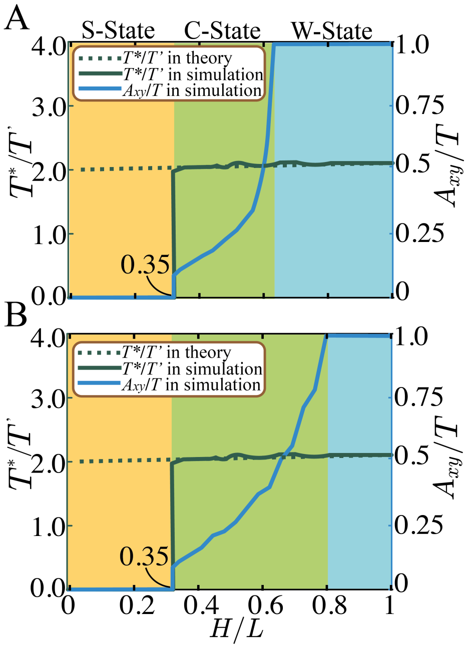

Figure S37: **The choice of elastic constant affects the defect state boundary**. Simulation results of 1D cusp-like splay-bend pattern ($\Psi = 12°$) using (A) 5CB elastic constants and (B) one-constant.

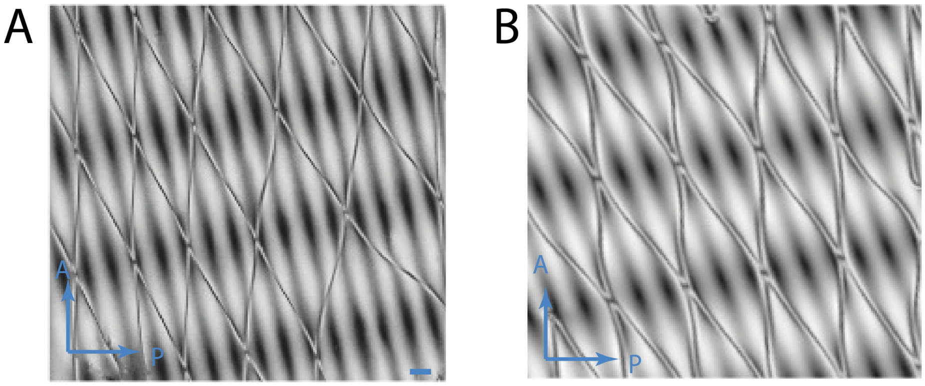

Figure S38: **Comparison of optical images.** Experimental optical image (A) and simulated optical image (using Nemaktis [8]) (B) in the W-state from the 1D cusp-like splay-bend pattern. Scale bar: $50\ \mu$m.

References

[1] Sunami, K., Imamura, K., Ouchi T., Yoshida, H., Ozaki, M., 2018. *Shape control of surface-stabilized disclination loops in nematic liquid crystals*. Physical Review E 97: 020701.

[2] Ouchi, T., Imamura, K., Sunami, K., Yoshida, H., Ozaki, M., 2019. *Topologically protected generation of stable wall loops in nematic liquid crystals*. Physical review letters, 123(9), 097801.

[3] Yoshida, H., Asakura, K., Fukuda, J. I., Ozaki, M., 2015. *Three-dimensional positioning and control of colloidal objects utilizing engineered liquid crystalline defect networks*. Nature communications, 6(1), 7180.

[4] Duclos, G., Adkins, R., Banerjee, D., Peterson, M.S., Varghese, M., Kolvin, I., Baskaran, A., Pelcovits, R.A., Powers, T.R., Baskaran, A. and Toschi, F., 2020. *Topological structure and dynamics of three-dimensional active nematics*. Science, 367(6482): 1120-1124.

[5] Binysh, J., Kos, Ž., Čopar, S., Ravnik, M. and Alexander, G.P., 2020. *Three-dimensional active defect loops.* Physical Review Letters, 124(8), 088001.

[6] Jiang, J., Ranabhat, K., Wang, X., Rich, H., Zhang, R. and Peng, C., 2022. *Active transformations of topological structures in light-driven nematic disclination networks*. Proceedings of the National Academy of Sciences, 119(23), e2122226119.

[7] Amidror, I., 2009. *The Theory of the Moiré Phenomenon: Volume I: Periodic Layers (Vol. 38)*. Springer Science and Business Media. See 3.4.1 for the theory of two superposed square lattice.

[8] Guilhem Poy. Nemaktis. https://github.com/warthan07/Nemaktis.

[9] Alexander, G.P., Chen, B.G.G., Matsumoto, E.A. and Kamien, R.D., 2012. *Colloquium: Disclination loops, point defects, and all that in nematic liquid crystals*. Reviews of Modern Physics, 84(2), p.497.

[10] De Gennes P. G., Prost J. 1993. *The physics of liquid crystals*. Oxford university press.

[11] Stewart, I. W. 2019. *The static and dynamic continuum theory of liquid crystals: a mathematical introduction*. CRC Press.

[12] Ondris-Crawford, R., Boyko, E.P., Wagner, B.G., Erdmann, J.H., Žumer, S., Doane, J.W., 1991. *Microscope textures of nematic droplets in polymer dispersed liquid crystals*. Journal of applied physics, 69(9): 6380-6386.

[13] Ellis, P.W., Pairam, E., Fernández-Nieves, A., 2019. *Simulating optical polarizing microscopy textures using Jones calculus: A review exemplified with nematic liquid crystal tori*. Journal of Physics D: Applied Physics, 52(21), 213001.

[14] Yeh, P., Gu, C., 2009. *Optics of liquid crystal displays*. John Wiley and Sons.

[15] Mauguin, C., 1911. *Sur les cristaux liquides de M. Lehmann*. Bulletin de Minéralogie, 34(3), pp.71-117.

[16] Chen, J., Akomolafe, O.I., Dhakal, N.P., Pujyam, M., Skalli, O., Jiang, J. and Peng, C., 2022. *Nematic templated complex nanofiber structures by projection display*. ACS Applied Materials and Interfaces, 14(5): 7230-7240.
