# Supplementary material for: Moiré effect enables versatile design of topological defects in nematic liquid crystals
Source: Nat Commun. 2024 Feb 26;15:1655. doi: 10.1038/s41467-024-45529-z (PMC10897219; doi:10.1038/s41467-024-45529-z)
Supplement: Supplementary file 3 — Description of Additional Supplementary Files [file 41467_2024_45529_MOESM3_ESM.pdf]

Description of Additional Supplementary Files

File Name: Supplementary Movie 1
Description: Simulated nematic moiré pattern from 1D cusp-like splay-bend pattern. We increase the cell gap H while fixing the lattice constant L and rotation angle at $\Psi = 12°$. Gap-to-pattern ratio H/L is varied between $[0.1, 0.83]$. Left: defect structure gradually changes from the S-state, to the C-state and the W-state; yellow short lines represent director field in the $z = H/2$ plane. Right: two identical geometric lattices of the same L as the nematic surface pattern are superposed; this can give rise to the same moiré period T as the defects.

File Name: Supplementary Movie 2
Description: Simulated optical pattern by rotating crossed polarizer and analyser. For a nematic moiré formed from 1D cusp-like pattern in the C-state with $\Psi = 11°$ and $H/L = 0.5$, we simultaneously rotate the crossed polarizer and analyser by 90°, and find that the grain-like optical pattern is insensitive to the choice of the orientation of the cross polarizers.

File Name: Supplementary Movie 3
Description: Layer-by-layer scanning of the W-state defect structure using confocal microscopy. The scanning goes from the top layer of the cell to the bottom. The movie starts with the layer of the 1st group of disclination and ends where the second group of defect lines shows up.

File Name: Supplementary Movie 4
Description: 3D rotational view of the W-state defect structure using confocal microscopy. The web-like disclination consisting of two defect groups can be observed. Note that disclination lines close to the top substrate scatter the laser light so that their signal extends from one surface to the other.

File Name: Supplementary Movie 5
Description: Experiments of transportable and reconfigurable colloidal assembly. POM results with scale bar $= 50$ μm. As Ψ increases, the reorganization of defect lines enables the well-controlled assembly of colloidal chains. The process is reversible.

File Name: Supplementary Movie 6
Description: Simulated periodic defect structure formed from a 1D cusp-like splay-bend pattern. The rotation angle Ψ is varied from 1° to 45° at fixed $H/L = 0.67$.

File Name: Supplementary Movie 7
Description: Experiments of the nematic moiré from the 1D cusp-like splay-bend pattern by varying rotation angle Ψ. POM results with scale bar $= 50$ μm. The system starts with a defect-free state. As Ψ increases, the number of defect lines increases and their orientations tilt, confirming the simulations.

File Name: Supplementary Movie 8
Description: Experiments of 3D network colloidal assembly. POM results with scale bar $=$ 50 μm. Colloidal particles are attracted to two groups of defects and assembles into a 3D network. Rotation angle is $\Psi = 50°$.

File Name: Supplementary Movie 9
Description: Emergence, annihilation and reconfiguration of defect loops formed from 1D sinusoidal splay-bend pattern in simulations. Defect loops are generated by relatively twisting two identical 1D sinusoidal splay-bend anchoring patterns of a nematic cell by $\Psi = 30°$. The left is a top view and the right is a angled view with the mid plane director and the defects in 3D. The group of defect loops with the smaller size annihilates as the cell gets thicker. Eventually, all loops become web-like line defects and are suspended near the two surfaces, leaving a uniform director filed in the mid plane. L is fixed while increasing H/L from 0.08 to 1.

File Name: Supplementary Movie 10
Description: Simulated defect structure transformation during rotation operation over a 1D sinusoidal pattern. Ψ is varied from $6°$ to nearly $90°$. Defect loops period T decreases as Ψ increases. Meanwhile, defect loops transition from elongated shapes to more round shapes. The left is LC defect structures colored by z/H; the right is the corresponding geometric moiré with the same lattice constant L.

File Name: Supplementary Movie 11
Description: Simulated defect structures from 2D ± 1 defect pattern. The rotation angle Ψ is varied from 1° to 45°. The left is the defect structure in a 17 by 17 ± 1 defect lattice; the right is the corresponding 17 by 17 geometric moiré. For $\Psi \leq 21°$, they show the same periodicity and tilting angle.

File Name: Supplementary Movie 12
Description: Simulated defect structure from 2D anchoring pattern responding to translation operation showing the feature of conventional moiré pattern. The nematic moiré from 2D lattice of ± 1 surface defects is firstly relaxed at $\Psi = 5°$, then the top substrate is translated horizontally, upon which the disclinations exhibit a vertical shift. This shows the topological structures in nematic moirés carry the same feature of isotropic moirés.

File Name: Supplementary Movie 13
Description: The effect of rotation speed. For rotation rate 1, the dimensionless time for a π-turn is $\tau_s/\tau_{\text{cell}} = 35.4$. For rotation rate 2, the dimensionless time for a π-turn is $\tau_s/\tau_{\text{cell}} = 236$. The periodic defect structure is vanished for the high rotation rate, and is preserved for the low rotation rate.

File Name: Supplementary Movie 14
Description: Simulated electric field induced transition in the nematic moiré from the 1D cusp-like splay-bend pattern. Before the electric field is turned on, the system is in the S-state. The field is applied along the cell height (z direction). From the middle plane director field evolution, out-of-plane rotation of the director field is initiated in larger twist regions (in between neighboring defect curves).
